# Characterizing the behavioural correlates of error awareness with the novel speeded inference task

**DOI:** 10.1038/s41598-026-56114-3

**Published:** 2026-06-03

**Authors:** Eva Niessen, Jonas Wickert, Martin Schober, Gereon R. Fink, Peter H. Weiss, Jutta Stahl

**Affiliations:** 1 Department of Individual Differences and Psychological Assessment, University of Cologne, Cologne, Germany 50969; 2 Cognitive Neuroscience, Institute of Neuroscience and Medicine (INM-3), Research Centre Juelich, Juelich, Germany; 3 Structural and functional organisation of the brain, Institute of Neuroscience and Medicine, Research Centre Juelich, INM-1), Juelich, Germany; 4 Department of Neurology, University Hospital Cologne, Cologne, Germany

**Keywords:** Error processing, Performance monitoring, Experimental design, Cognitive control, Psychology, Human behaviour

## Abstract

Influential theories on error processing assume that when we make errors, adaptive processes are triggered to improve our behaviour. These processes appear to be more effective after participants have detected an error. Therefore, the assessment of error awareness, allowing a differential analysis of detected and undetected errors, and behavioural adjustments have gained increasing attention in the field of cognitive control over the past decades. A common methodological challenge in studies investigating error detection is that the number of undetected errors is usually relatively low. Here, we introduce a new experimental task that pursued the primary goal to generate a high error rate including many detected and undetected errors which is stable over the time on task. Study 1 (n = 21 adults) clearly showed the task was successful in producing those indented error rates. Exploratory analyses of study 1 further suggested that post-error adjustments (measured as post-error slowing and post-error accuracy) are unrelated to error awareness in the current study. Based on findings of study 1, study 2 (n = 20 adults) used the experimental task to test whether error awareness could specifically be manipulated within one experimental session by changing a single task feature. As expected, the modulation affected error detection, but did not affect the total error rate. Potential applications of the Speeded Inference Task (short SIT) are discussed. With this newly developed paradigm, we wish to lay the foundation for future research to better understand (neural) processes associated with error awareness.

**Supplementary Information:**

The online version contains supplementary material available at 10.1038/s41598-026-56114-3.

## Introduction

The assessment of error awareness in the context of cognitive control and behavioural adjustments is essential and has gained more and more attention in the past decades. Research questions in this field are usually investigated by a combination of brain activity (e.g., electroencephalography, EEG) and behavioural response parameter (e.g., RT, error rates)^1–3^. Influential theories on error processing assume that when we make errors, adaptive processes are triggered to improve our behaviour and prevent errors in the future^4,5^. These processes occur even when we are unaware of the incorrect action^6,7^, but they appear to be more effective after aware than unaware errors^8^. Commonly used measures of behavioural adaptations after errors are slowing of response times (post-error slowing, PES) or increases in accuracy (post-error accuracy, PEA)^9^. While PEA represents an immediate performance improvement, the functional significance of PES is still under debate. PES might indicate an adaptive adjustment (e.g., a more cautious, hence slower, response in the trial following an error to increase the chance of stopping or changing the action successfully;^7,10^. Alternatively, PES could merely represent a (maladaptive) by-product of the occurred error (e.g., processing the infrequent error results in a delay or disruption of the forthcoming processes resulting in slower responses in the trial following an error;^11,12^.

One methodological challenge posed on all studies investigating error processing is to employ an experimental task that results in many errors for all participants (i.e., a high error rate). A gold standard for excluding participants from error-related analysis (especially relevant for EEG studies) is a cut-off below six error trials based on reliability analyses^13,14^. Even though this might not sound much, post-hoc reductions in sample size are often problematic in studies examining neural parameters of error processing but also for the post-error behavioural parameter. In the past, several methods have been used that increase task difficulty and consequently the error rate (e.g., reduce stimulus contrast to induce perceptual conflict or increase the number of distracting stimuli;^15,16^. However, caution is advised because too many errors due to high task difficulty might lead to frustration and disengagement from the task and whenever participants disengage from a task, they no longer process their errors thoroughly^17,18^. Besides this issue, it is important to consider that, if studies investigate error awareness, it is not only necessary to generate many errors in each participant, but ideally many detected and undetected errors, because bases on the research question the error types will be preferably analysed separately. A quite balanced number of the two error types would also be helpful for contrasting the conditions using specific statistical models on EEG data (e.g. multivariate pattern analyses^19^) or behavioural data (e.g., computational modelling^20^ ) to investigate the underlying mechanisms properly. In reality, however, the number of undetected errors is usually very low^3,21–23^. Please note that we want to clarify that when we use the term *error awareness*, we refer to the conceptual process of being conscious of the incorrect action. In contrast, when we use the term *error detection*, we refer to the operationalisation of awareness in the sense that participants volitionally reported their awareness. Past attempts to provoke more undetected errors, for instance, reduced the visibility and processing time of the stimulus^24,25^, leading to perceptual uncertainty and reduced error detection rates^26^. Another approach was to increase task duration, resulting in fatigue and more undetected errors, especially at the end of the experimental session^27^. Yet, as stated above, errors due to fatigue differ from errors due to other sources with respect to the underlying cognitive processes. Thus, to better understand the underlying mechanisms as well as influences of error awareness on the performance, it is essential to implement tasks that produce many errors, especially many undetected ones, without causing a lack of motivation, frustration, fatigue or an (almost) unsolvable task.

While the above-described procedures indeed resulted in a larger number of undetected errors, it is questionable whether the participants in the first place could have adequately detected the observed errors. Rather, these experimental manipulations most likely prevented adequate evidence accumulation^28^, i.e., the collection of sufficient (sensory) information about a stimulus that is necessary for deciding on the correct response. Thus, a prerequisite for participants to become aware of their errors is accumulating enough evidence and generating a mental representation of the correct response^29,30^. Thus, the applied methods may have triggered mere guessing. If so, differences between the processing of detected and undetected errors would happen by chance rather than result from differential underlying mechanisms. Support for the hypothesis that undetected errors are processed differently than detected errors comes from studies assessing measures of the autonomic nervous system (e.g., pupil dilation). These studies claim that undetected errors result from reduced task engagement and reduced autonomic reactivity compared to detected errors^12,31,32^.

In addition to the still unknown origin of the emergence of error awareness, behavioural consequences are likewise unknown. While it might be intuitive to expect stronger adjustments after detected errors, findings are inconsistent^9^. Results of previous studies might be difficult to compare due to, for example, differences in task design, but also, the choice of measure for behavioural adjustments matters. For instance, Kirschner and colleagues (2021) report an effect of error awareness on PES, but no effect on PEA. Likewise, Dali, Orr and Hester (2022) found a weak association between PES and error awareness without any influence on PEA.

A final aspect that needs to be considered is the participants’ motivation *not* to commit errors. If errors are irrelevant for participants, the motivation to adapt one’s behaviour is usually low^33^. Meaningful errors are coupled with emotional appraisal, and the affective response triggered by errors is assumed to result in more robust adaptive processes (e.g., larger PES^34)^. One of many ways to increase motivation is by making experimental tasks more appealing to participants^35^. A recent successful attempt showed that for instance a gamified stop-signal task produced a similar behavioural pattern as a classical stop-signal task, but with elevated motivation levels^36^. As this study did not examine error awareness and further evidence is sparse, it remains unknown whether and how motivation is related to error awareness. A first hint that high motivation is also relevant for error awareness comes from Di Rosa and colleagues (2021), who compared the performance on a standard error awareness task to a motivational version and unexpectedly found that in the motivational task, participants made significantly more errors, which were (at least descriptively) more often undetected.

## Objectives

Here, we introduce a new experimental paradigm for assessing error awareness that aims to overcome some of the described methodological limitations. The benefits and characteristics of the paradigm will be described in detail, making it easy to use and adjustable to individual needs. Several features were implemented to make the testing situation appealing to participants, hoping to increase their intrinsic motivation. Those features aimed at a challenging but achievable task and included amongst others a realistic set of stimuli (e.g., birds or ice cream rather than abstract geometric forms), relatively simple instructions (e.g., participants only had to apply one of two hierarchical rules), an individually adjusted task difficulty and a reward system.

The overarching objective of the present work was to establish the new paradigm and inspect the resulting behavioural patterns, primarily focusing on the amount of detected and undetected errors. The first goal of study 1 was to proof that the speeded inference task (SIT) would be able to achieve a minimum of six trials per error type per participant to reach the necessary criterion for reliably investigating error processing in EEG research^13,14^. The second goal was to obtain a stationary error rate across the experimental session, which was intended to be achieved by an adaptive algorithm. Finally, in the first study, we explored highly relevant parameters in the field of error processing by means of post-error adjustments (i.e., PES and PEA) and their associations with error awareness. If researchers wish to use the SIT, it is important to know what patterns of post-error adaptation can be expected. The literature so far reported inconsistent results on error awareness and behavioural adaptation – some found adaptation after undetected errors while others did not^9^. With the potentially higher number of undetected errors, we aimed to identify potential correlations between the error detection rate and PES and PEA to improve our understanding of the functional/behavioural relevance of these post-error adjustments.

Based on descriptive results from study 1 showing that the error detection rate, but not the general error rate, was influenced by the presented rule, the major goal of study 2 was to test whether it was possible to specifically manipulate the error detection rate within one experimental session by changing a single task feature (the ratio of the two rules). If successful, this systematic manipulation would reveal further applications of our new paradigm for the research on error awareness.

### General methods

This study was performed in line with the principles of the Declaration of Helsinki. Approval was granted by the Ethics Committee of the Deutsche Gesellschaft für Psychologie (DGPs; reference number EN 042018). Before starting with the experiment, participants gave written informed consent. The paradigm and the experimental parameters of the two studies were identical, with one exception (i.e., rule frequency, see below). Thus, the following descriptions apply to both studies.

## Participants

We recruited young, healthy, right-handed (Edinburgh Handedness Inventory, EHI;^37^ ) participants without any history of neurological or psychiatric diseases via mailing lists amongst students and employees at the Research Centre Juelich. Participants were paid 10 € per hour for compensation. Task instructions and questionnaires were provided in German or English (i.e., participants had to be fluent in German or English). In addition, all participants stated to have intact colour vision.

For study 1, 21 participants were recruited (twelve female, nine male; mean age and standard deviation was 26.8 ± 3.8 years, ranging from 20 to 34 years). For study 2, we recruited a fresh sample of 20 participants (ten female, ten male participants; mean and SD age 25.1 ± 5.4 years, ranging from 18 to 35 years). All participants gave written informed consent. As the SIT is a novel task and the to-be achieved goals were descriptive in nature for study 1, a calculation of a sample size estimation was not possible. The sample size of study 2 was kept comparable to the sample size of study 1 to allow a fair comparison of the replication (i.e., part 1 of study 2).

## Experimental paradigm

**Fig. 1:**
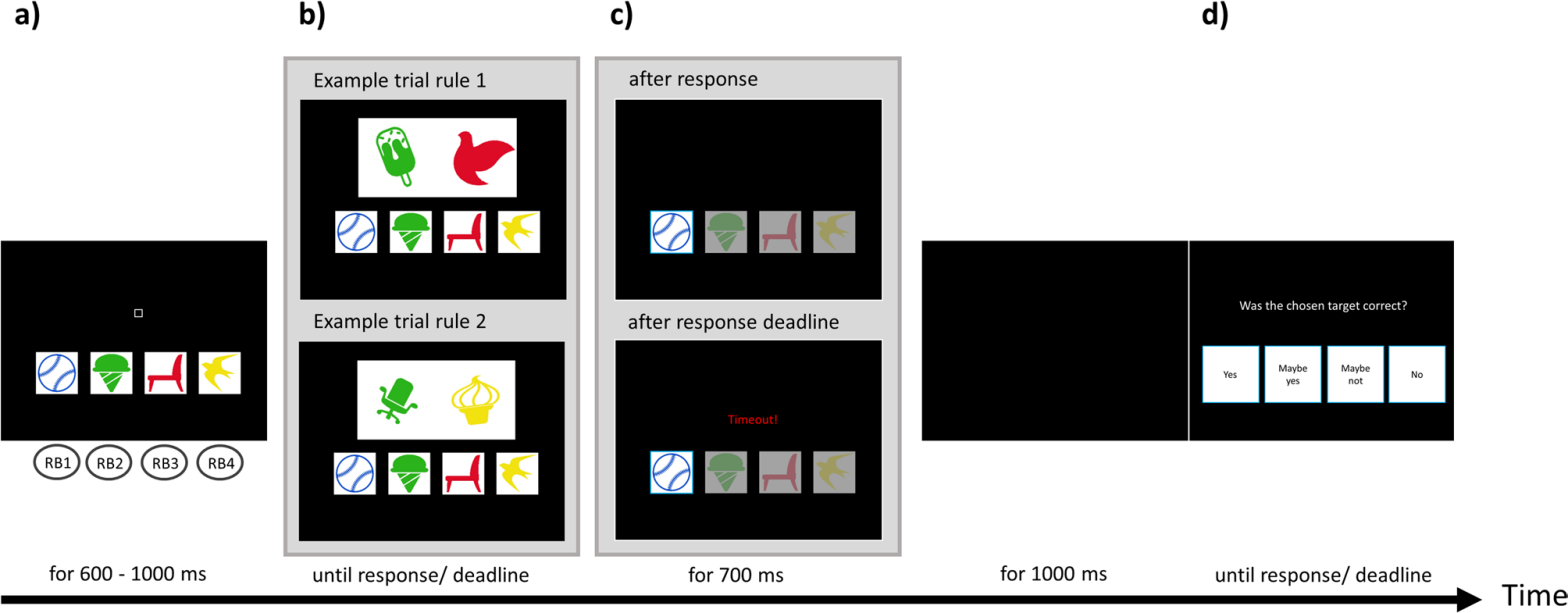
Display during the task and example trials. Only one of the displays included in B and C (inside grey background) are shown in each trial, but otherwise, the course was identical for all trials. (**a**) Four targets are shown constantly throughout the experiment (here: a blue ball, a green ice cream, a red chair, and a yellow bird). Targets should be chosen by pressing the corresponding response button (RB; left middle finger for the blue ball, left index finger for green ice cream, etc.). (**b**) On each trial, a stimulus appears above the targets representing two mixtures of the target dimensions. Rule 1 or rule 2 had to be applied depending on the stimuli. Top: Example of a trial applying rule 1, where the correct target would be the green ice cream because green ice cream is part of the stimulus. Bottom: Example of a trial applying rule 2, where the correct target would be the blue ball. One or more features of the other targets are present in the stimuli, but neither the colour blue nor a ball is present in the two stimulus items. (**c**) Top: After a response is given by selecting one of the targets, this target is highlighted (here: the blue ball, which is an error). Bottom: When no response is given until the deadline, the word ‘Timeout’ replaces the stimulus, and the software chooses a random target. The highlighting of the chosen target is identical to the top scenario and is followed by a black screen. (**d**) An evaluation screen asks participants to rate the previous decision on a four-point scale using the same four RB as in the main task.

**Task**. The experimental paradigm is a speeded hierarchical decision-making task with two stimulus dimensions and four response options – and is called the Speeded Inference Task (SIT)^1^. The goal for participants was to identify on each trial the correct target out of a constant set of four targets (ball, bird, ice cream, chair) based on specific rules (see below). The targets were mapped to four spatially congruent response buttons, which should be pressed with the left and right middle and index fingers (Fig. 1A). Thus, the most left target was assigned to the left middle finger, while the most right target was assigned to the right middle finger. The four targets were defined by a colour-category match that remained constant throughout the experiment. A possible target constellation is shown in Fig. 1 (blue ball, green ice cream, red chair, and yellow bird).

**Stimulus material**. The two presented stimuli were drawn from seven different exemplars of each target category. Thus, the participants had to respond to the concept, e.g., “ball”, and not to the specific stimulus exemplar. The four target exemplars were constant for all participants. They were deliberately chosen because they were most representative of that category. The 28 exemplars used for the stimuli were shown to the participants before the experiment to prepare participants for the stimulus diversity (see Supplementary Material 1). The objects in the stimuli were variable in size (reduction up to 60% possible compared to the picture’s original size) and orientation (tilted for ± 40 degrees). The stimuli changed in every trial in form, colour, size and inclination.

**Rule Types.** A stimulus appeared above the targets on each trial and showed two items representing a mixture of the target features (see Fig. 1B, examples for the two rules). Participants were instructed to apply two rules in a hierarchical order to identify the correct target based on a given stimulus. *The first rule* implies that if one of the two items represents the same category *AND* the same colour as one of the targets, this target should be chosen (see Fig. 1B top, example rule 1: green ice cream). If the first rule is not applicable, the participants should apply *the second rule*. In that case, participants should search for the target whose category and colour are both *NOT* represented in the two items of the stimuli, i.e., the target that differed in both colour and category when compared to the two stimulus items (see Fig. 1B bottom, example rule 2: blue ball). In each trial, the participants had to identify from the presented stimuli which of the two rules should be applied (i.e., there was no explicit cue indicating the appropriate rule was given).

**Performance evaluation.** After a target was chosen, participants were asked to evaluate the correctness of their decision. A rating screen appeared, asking “Was the chosen target correct?”. Participants could answer by using the same four response fingers. They could choose to answer “yes” (left middle finger) or “no” (right middle finger), and in addition, we allowed the expression of uncertainty in their evaluation and hence also provided the potential answers “maybe yes” (left index finger) or “maybe not” (right index finger, see Fig. 1D). The response was assumed to reflect the degree of error awareness resulting from continued internal evaluation of the trial, unbiased by any form of direct performance feedback.

**Timeout**. One build-in feature was a sudden abortion of a running trial. In these cases, before the participant responded, the stimulus was replaced by the word ‘TIMEOUT’ (written in red ink; see Fig. 1C bottom). Timeouts were signals for too-slow responses and in these cases, the software randomly chose one of the targets. This randomly chosen target could be correct or incorrect. Unbeknownst to the participants, the temporal threshold for timeouts was constantly adjusted based on the past accuracy of the participants (see “Further task features”).

**Trial course**. Figure 1 illustrates the sequence and time course of a trial. Each trial started with presenting the four targets and a small square representing a fixation point in the centre above the targets. After a random interval of 600–1000 ms, the stimuli appeared above the targets. The stimuli disappeared when a response was given, and for 700 ms, the chosen target was highlighted (Fig. 1C top) by a turquoise frame surrounding the chosen target, whereas the transparency of the three not-chosen targets was increased. This layout was identical for self-chosen targets and the automatically chosen targets after a timeout (additionally marked by the word ‘TIMEOUT’; Fig. 1C bottom). A black screen appeared for another 1000 ms before the rating screen to evaluate the response was presented. After participants had chosen an evaluation response or no response was given after 4000 ms, the inter-trial interval (see above 600–1000 ms) started, including the presentation of the targets and the fixation square.

## Procedure

In a quiet room with dimmed illumination, participants were seated in front of a black screen (LCD monitor, 60 Hz) at a distance of approximately 70 cm. Responses were measured with temporally precise response pads (LumiTouch, Burnaby, Canada). The experimental paradigm started with two training sessions, followed by the main task. The content of the self-paced instructions, by which participants were asked to respond as quickly and accurately as possible, can be found in Supplementary Material 2. It was assumed to be beneficial to practise the target-finger mappings in order to avoid errors due to difficulties in mapping targets and response fingers. Therefore, we included two training sessions, which could be repeated until participants felt comfortable, and the experimenter was satisfied with the performance (minimum was 8 trials per training session). More specifically, participants were only allowed to continue when they showed automation using the four response buttons (i.e., learned the target-response mapping) and when they were not frequently interrupted by timeouts (i.e., they responded as quickly as desired). The first training session was mainly intended to introduce participants to the task layout and familiarise them with the stimuli and targets. There was no speed limit, and trial-wise feedback should help to become acquainted with the task and the two rules. The second training session resembled the main task (i.e., no feedback was given, and the timeout was used); only the timeout threshold was not adjusted. On average, one experimental session (including instruction, experimental task, questionnaires and debriefing) lasted between 50 and 60 min.

## Further task features

**Adaptive algorithm**. The adaptive algorithm was implemented to keep the difficulty level comparable across participants and avoid ceiling or floor effects. The threshold for timeouts started at 2000 ms. The threshold was adjusted after 16 trials (thus twice per block). When the average accuracy of the last 16 trials was below 60%, the threshold was increased by 10%. When the average accuracy of the last 16 trials was higher than 90%, the threshold was reduced by 10%. The timeout threshold remained unchanged when accuracy lay between 60 and 90%. Please note that it was not the intention to obtain a fine-tuned error rate (e.g., as could be gained by a staircase procedure), but rather to have a comparable and high error rate per participant. Participants were not informed about the temporal adjustments.

**Scoring System**. Another feature was a scoring system that provided participants with an indication of their performance during the breaks between the blocks. Notably, we avoided trial-based feedback as behavioural adaptation should not rely on external feedback. Participants could gain 10 points for each correct response on the main task and a further 3 points for correct evaluations. A good performance on the main task was most important, but the evaluations also required the participants’ attention. The points for the correct evaluations were given independent of the certainty of the evaluation (e.g., the answers ‘yes’ and ‘maybe yes’ as well as ‘no’ and ’maybe no’ were treated identically). Importantly, participants were instructed to also rate the automatically chosen target in timeout trials. Thus, participants could earn 3 points in such a trial by correctly evaluating the randomly chosen target. This allowed us to assess the participants’ awareness of these automated decisions. During breaks, participants were presented with the maximum score that could have been reached up to that moment and the score participants had reached. Further, participants were informed about a guaranteed reward for the best participant in the current sample. Therefore, the final score of the best participant was shown in all breaks to increase the motivation of the participants to beat this highscore. The intention was to prevent fatigue and boredom at the end of the experimental session and obtain a good performance throughout the entire session.

**Controlling procedures**. We balanced the target colour-category matching across participants, precluding an unwanted colour-based bias (e.g., red could attract more attention); thus, each colour could be assigned to a different finger. The categories were always allocated to the same spatial positions.

Further, the correct target-response assignment frequency was equally distributed within each block; thus, within one block (32 trials), each of the four targets was the correct response eight times. Perfectly balancing the timeout displays was impossible as the number of timeouts varied between participants. We defined a set of eight targets (each target twice), and in case of a timeout, the displayed target was drawn randomly (without replacement) from this set – thus controlling for a similar number of chosen targets independent of the correctness of that target in the given trial. When more than eight timeouts occurred, the set was filled again with eight targets, and the random drawing began anew.

### Study 1

#### Methods - specifics of study 1

In Study 1, the task consisted of 14 experimental blocks with 32 trials (i.e., 448 trials in total). Between each block, there was a break with a self-chosen duration (minimum break duration was 3 s). Both rules had to be applied similarly often within each block, hence 50% of the time.

**Data and statistical analysis.** The general response rates are going to be presented on a descriptive level. To investigate the changes in the different response rates across time, repeated-measures analyses of variance (RM ANOVA) were employed with the within-subject factor block (block 1–14) and as dependent variables, either correct rate, error rate, timeout rate, or error detection rate. The correct rate, error rate, and timeout rate correspond to the proportion amongst all trials that were responded to either correctly, incorrectly, or where a timeout had occurred. For the calculation of the error detection rate (number of detected errors/ all errors), detected errors were defined as those errors which were rated as being incorrect (i.e., rating question ‘Was the chosen target correct?’ answered with ‘No’ and ‘Maybe not’). Undetected errors, consequently, were those errors rated as being correct (i.e., rating question ‘Was the chosen target correct?’ answered with ‘Yes’ and ‘Maybe yes’). For the influence of rule type, we conducted paired-sample t-tests for the error rate comparing rule 1 versus rule 2 trials [i.e., number of errors for rule 1 *or* 2 / 224 (224 is the sum of trials applying one rule type)]. The same was done for the error detection rates (e.g., number of detected errors for rule 1 / number of all rule 1 errors). Then, to assess a potential differential performance improvement due to the two rules, we conducted two 2 × 14 RM ANOVAs with the factors rule and block for the dependent variables error rate and error detection rate.

Response times (RT) were examined with a paired-sample t-test comparing RT of errors and correct responses. RT changes throughout the experiment were tested with a 2 × 14 RM ANOVA with the factors accuracy and block. Finally, RT differences for rule 1 and rule 2 trials were tested with a 2 × 2 RM ANOVA with the factors rule and accuracy.

Behavioural adaptations were assessed regarding post-error slowing (PES) and post-error accuracy (PEA). We followed a recent recommendation^53^ to base the calculation of PES on the absence or presence of RT fluctuations in the dataset. Using the Augmented Dickey-Fuller Test, we assessed in each participant whether the RT data followed a trend over time. RTs did not change significantly across time-on-task in any participant (the Augmented Dickey-Fuller Test was significant for all participants, all *p* < .05). Therefore, following the recommendations by Pfister and Foerster, PES was calculated by the traditional method, i.e., mean post error RT, while post-correct slowing (PCS) was defined as mean post correct RT^38,53^. Only those post-response trials were included, in which no timeout occurred (otherwise, there would be no RT on that trial). Paired-sample t-tests were conducted to compare PES with PCS. In addition, we investigated the influence of error detection on PES by splitting errors into detected and undetected errors and compared the respective PES using a paired-sample t-test. The same logic was applied to the analysis of PEA. First, the accuracy after errors (PEA) was statistically compared to the post-correct accuracy (PCA) using a paired-sample t-test, followed by a differentiation of PEA into PEA after detected errors and PEA after undetected errors.

## Results study 1

Unless stated otherwise, all results are expressed as the mean ± standard deviation (SD). Statistical tests were Bonferroni-corrected if applicable, and the Greenhouse-Geisser correction was used when sphericity was violated. Missing values were excluded from the analysis. Reported effect sizes are Cohen’s d (d) for paired t-tests and generalized eta-square (η²_G_) for repeated measures analyses.

The mean task duration was 37.4 ± 2.8 min and varied individually because trial durations varied with performance (e.g. timeouts, breaks). Here, we focus on correct and error responses. For more information about timeouts (e.g., descriptive statistics, behavioural adjustments after timeouts), please see Supplementary Material 3a.

### Response rates

**Descriptive statistics.** As shown in Table 1, the mean error rate was 18.3% (which, in absolute terms, means, on average, 82 error trials per person, range 51–144 errors), and few trials were disrupted by a timeout (8.7%). In only 4% of all ratings, one of the *maybe* options were used. Therefore, for the definition of detected and undetected errors, we collapsed the ‘Yes’ and ‘Maybe yes’ ratings to represent undetected errors and the ‘Maybe not’ and ‘No’ ratings for detected errors^39^. As a result, the mean error detection rate was 69.1% (meaning, on average, 27 undetected errors per participant, range 7–95; and, on average, 55 detected errors, range 33–87). Participants were able to reliably evaluate their correct responses, which was reflected in a very high correct detection rate^2^ of 98.2 ± 2.9%.

**Table 1:** Descriptives of task performance for studies 1 and 2.

	Correct (%)		Error (%)		Timeout (%)		Detection (%)
Overall Exp. 1	73.0 ± 3.9 [71.2–74.8]		18.3 ± 5.6 [15.8–20.9]		8.7 ± 4.0 [6.9–10.5]		69.1 ± 15.2[62.1–76.0]
Exp.1 Part 1 (50% rule 1)	67.7 ± 4.1 [65.7–69.9]		18.7 ± 7.6 [16.7–23.2]		13.6 ± 6.8 [9.6–14.9]		65.6 ± 15.7[57.5–73.4]
Overall Exp. 2	76.1 ± 3.1 [74.7–77.4]		15.5 ± 6.1 [12.8–18.1]		8.4 ± 5.1 [6.2–10.7]		68.4 ± 15.5[60.9–75.9]
Exp. 2 Part 1 (50% rule 1, baseline)	71.0 ± 7.0 [67.7–74.3]		16.3 ± 8.1 [12.5–20.1]		12.7 ± 7.0 [9.4–15.9]		63.8 ± 22.2[53.5–74.2]
Exp. 2 Part 2 (25% rule 1)	73.8 ± 3.9 [71.9–75.6]		17.6 ± 6.2 [14.7–20.5]		8.7 ± 6.2 [5.8–11.5]		76.3 ± 19.1[67.4–85.2]
Exp. 2 Part 3 (75% rule 1)	83.4 ± 3.7 [81.7–85.1]		12.5 ± 5.7 [9.9–15.2]		4.0 ± 4.2 [2.1–6.0]		63.5 ± 21.8[53.3–73.7]

Group mean ± SD are shown. Square brackets include the 95% confidence interval.

Detection = error detection rate (i.e., amount of detected errors/ all errors).

**Fig. 2:**
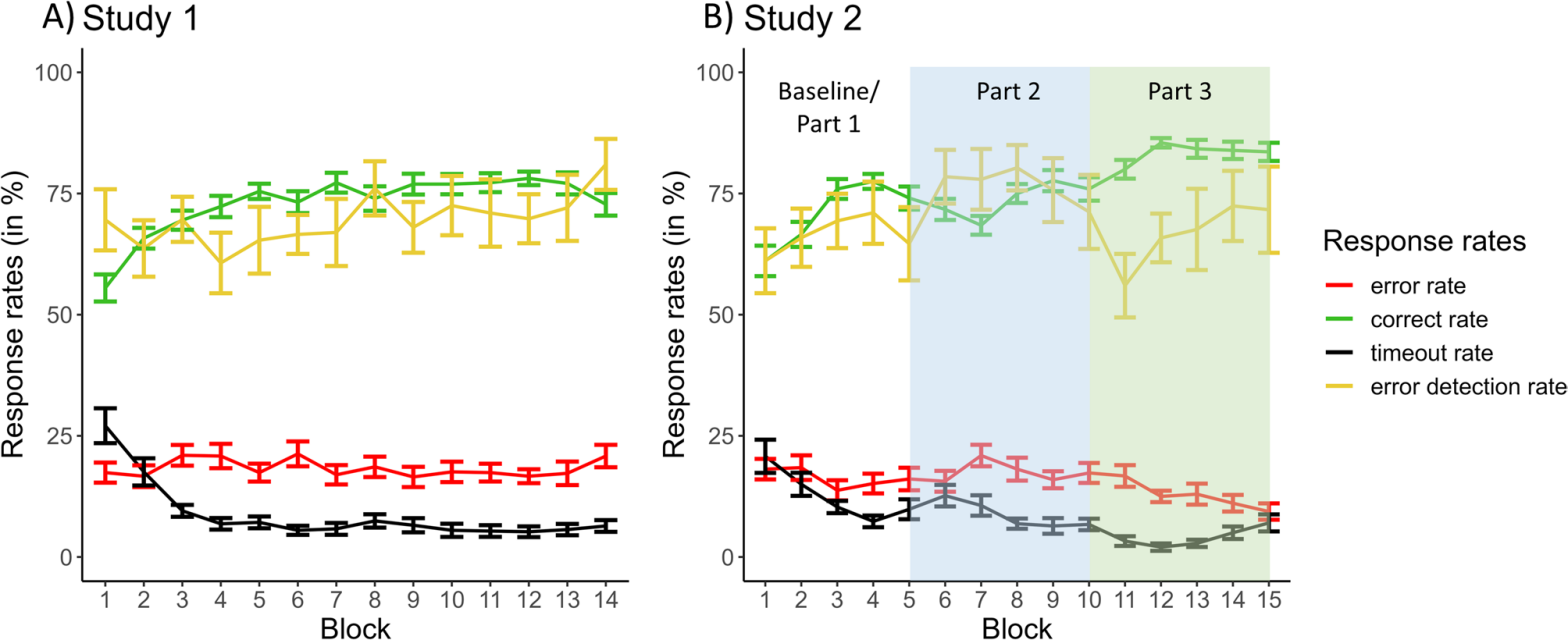
Mean ± standard error of the four response rates for each block of study 1 (**A**) and study 2 (**B**). Green line = correct rate; red line = error rate; black line = timeout rate; yellow line = error detection rate.

**Response rate variations across blocks.** First, we tested the effectiveness of the applied adaptive algorithm, which was based on the aggregated previous accuracy of the participants. The error rate did not differ across blocks throughout the task (Fig. 2A, red line). This was confirmed by the RM ANOVA, yielding a non-significant effect of the factor block [F(13,260) = 1.002, *p* = .449, η²_G_ = 0.033]. Intriguingly, the detection of errors was also unchanged across the experiment [Fig. 2A, yellow line; F(13,260) = 1.152, *p* = .317, η²_G_ = 0.043]. On the other hand, it was evident that participants’ performance got better with time, illustrated by a significant increase in correct responses [Fig. 2A, green line; F(13,260) = 8.906, *p* < .001, η²_G_ = 0.274], which could be explained by a reduction in the occurrence of timeouts throughout the course [Fig. 2A, black line; F(13,260) = 17.370, *p* < .001, η²_G_ = 0.389].

**Influence of rule type.** We expected that errors made on trials demanding the rule 1 and rule 2 could potentially differ in terms of behavioural response pattern. Therefore, we compared the number of errors that occurred when rule 1 should have been applied with errors occurring in trials when rule 2 had to be applied. Interestingly, the error rates did not differ significantly for the two rules [errors when rule 1 applied = 43.8 ± 16.7%, errors when rule 2 applied = 56.2 ± 16.7%; t(20) = -1.70, *p* = .106, d = − 0.370]. We observed great individual differences in the error rates split according to the two rules (see Supplementary Material 4), but these eventually balanced out on the group level. In terms of error detection, the two rules differed substantially [t(20) = -7.220, *p* < .001, d = -1.58]: While most errors on rule 2 were detected (87.1 ± 16.0%), the rating was at chance level for errors on rule 1 (48.7 ± 16.8%). Importantly and in contrast to the error detection rate, there was no difference in the correct detection rate between the two rules [99.1 ± 1.4% and 97.2 ± 5.9%, respectively for rule 1 and rule 2; *t*(20) = 1.488, *p* = .152]. This supports the specific role of awareness for errors, because the awareness of correct responses was not influenced by rule type.

**Fig. 3:**
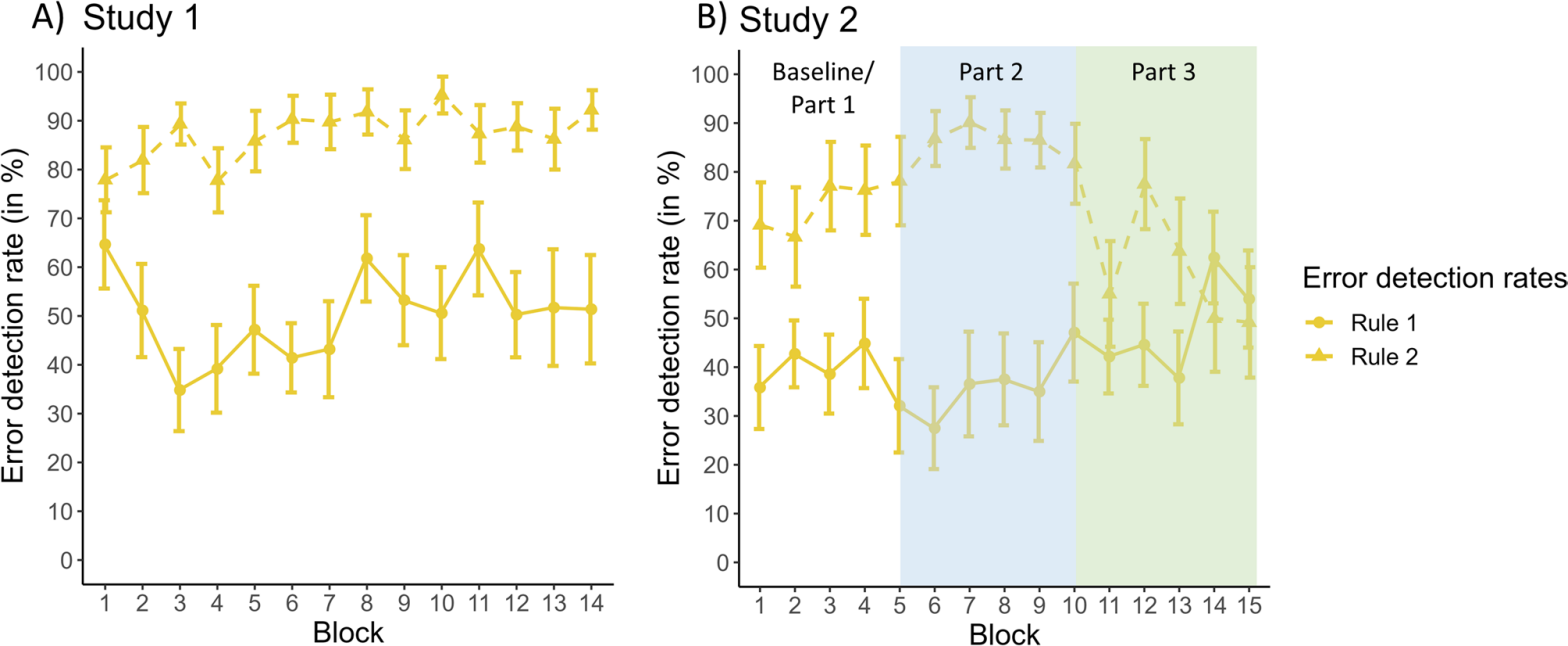
Detection rates of errors (mean ± standard error) conducted at trials demanding rule 1 (solid line) and rule 2 (dashed line) are shown for study 1 (**A**) and study 2 (**B**). In study 1, these did not change across the course of the experiment. Mean detection of errors in rule 2 was better than for errors in rule 1.

We further explored this by assessing changes in error rate and error detection rate for the two rules separately across the experiment. For the error rate in general, the 2 × 14 RM ANOVA with the factors rule and block did not show a differential development of the two rules [non-significant interaction: F(13,260) = 1.070, *p* = .297, η²_G_ = 0.014]. Regarding error detection, the RM ANOVA revealed only a significant main effect of rule due to higher error detection rates for errors on rule 2 than rule 1 [F(1,488) = 171.816, *p* < .001, η²_G_ = 0.260]. There was no significant main effect of block [F(13,488) = 0.912, *p* = .541, η²_G_ = 0.024], indicating no general change in error detection. Finally, there was also no significant interaction [F(13,488) = 1.061, *p* = .391, η²_G_ = 0.027], meaning that even though the detection of errors on the second rule was generally better, this did not result in a differential change over the experiment (see Fig. 3A).

### Response time

**Fig. 4:**
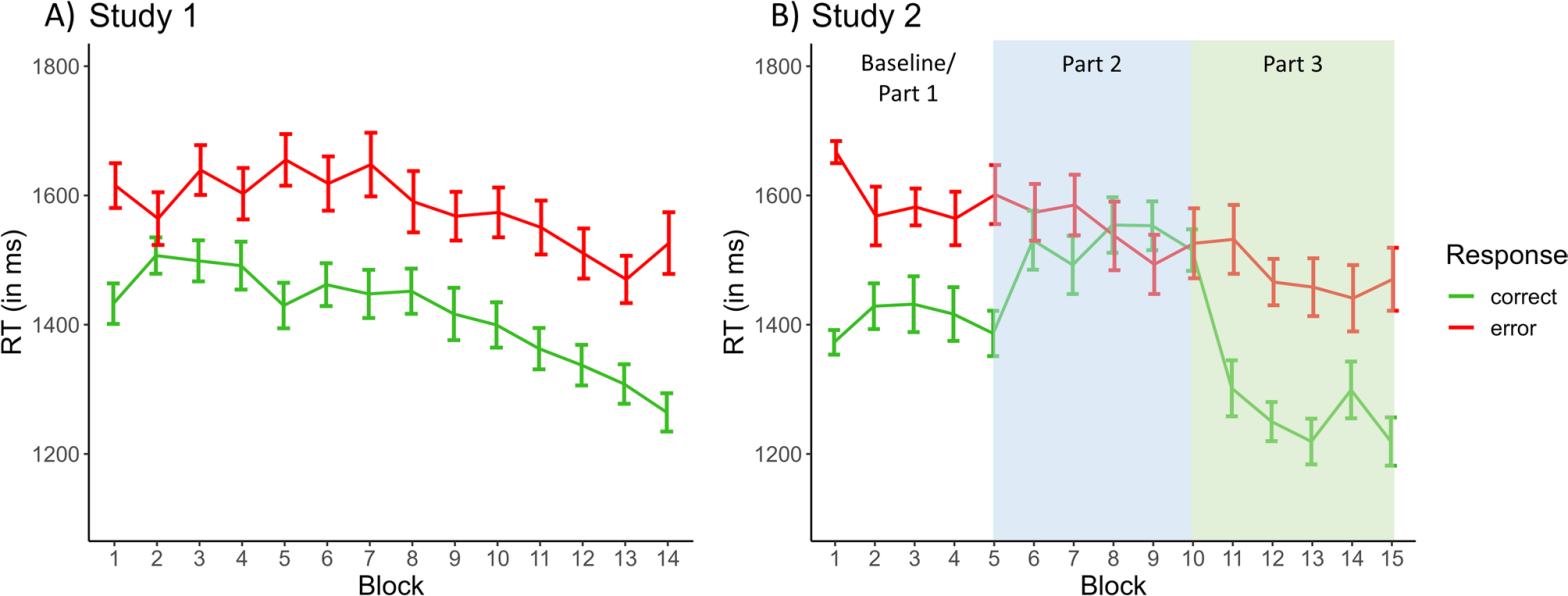
Response times (mean ± standard error) for errors (red line) and correct responses (green line) across all blocks are shown for study 1 (**A**) and study 2 (**B**). Generally, RTs are slower for errors than correct responses and are modulated by the rule frequency manipulation in part 2 of study 2.

The RT of errors was significantly slower than RT of correct responses [correct = 1398.5 ± 123.9 ms; error = 1672.4 ± 157.1 ms; t(20) = -15.763, *p* < .001, d = -3.44]. When inspecting the development of RTs across the experiment, the RM ANOVA revealed a main effect of accuracy [F(1,20) = 257.254, *p* < .001, η²_G_ = 0.346] and a significant main effect of block [F(13,260) = 7.327, *p* < .001, η²_G_ = 0.118]. This is in line with our interpretation above, arguing that participants became better on the task by avoiding timeouts and decreasing overall RTs. However, there was no significant interaction between block and accuracy [F(13,260) = 2.110, *p* = .150, η²_G_ = 0.017], indicating that the RTs became faster similarly for errors and correct responses (see Fig. 4A). For information about how RT was related to accuracy and rule type, please see Supplementary Material 3b.

### Behavioural adjustments

**Fig. 5:**
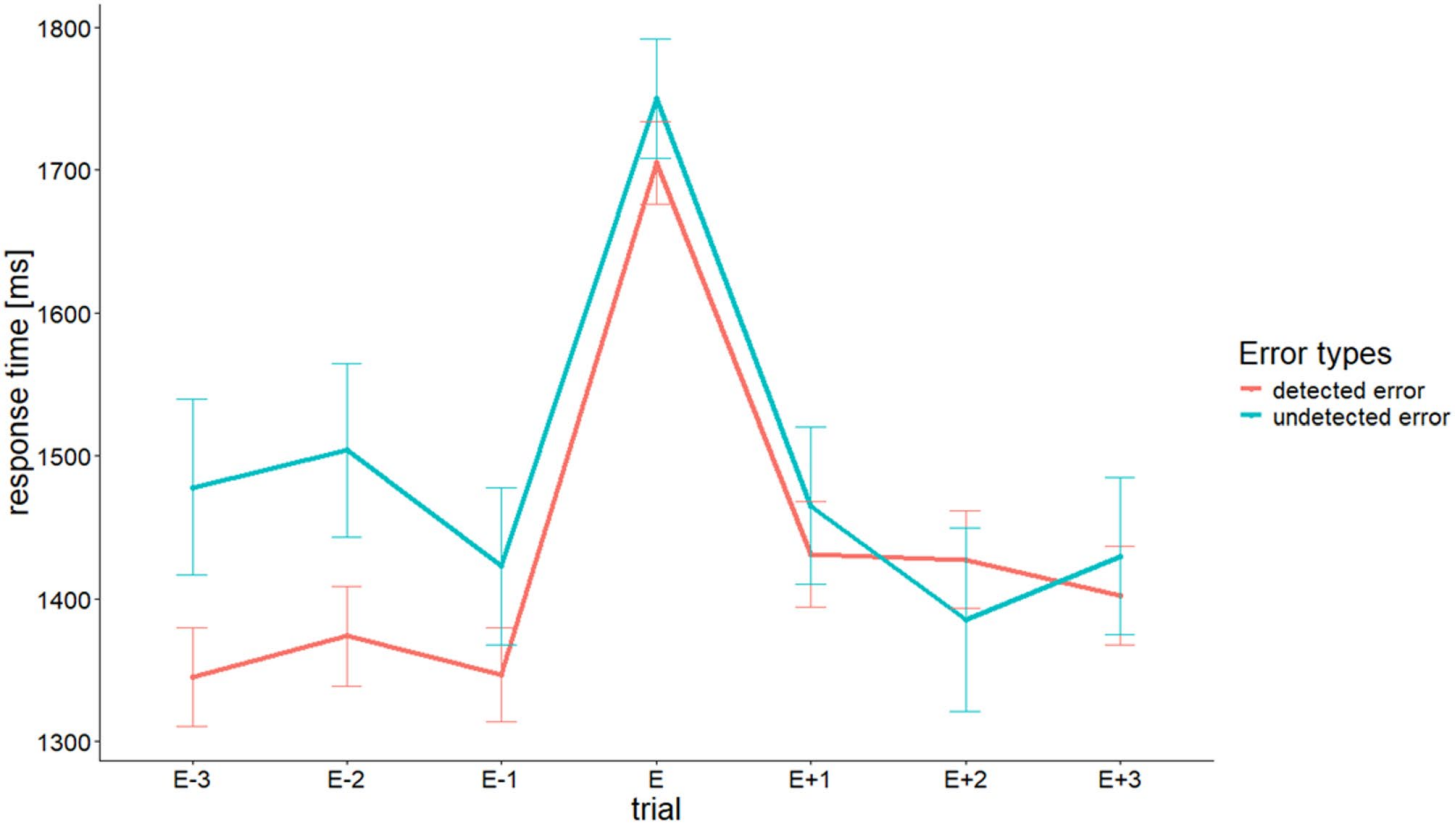
Post-error slowing is represented for detected and undetected errors. Mean ± standard error of response times of three pre- and post-error trials are plotted. Those trials were all correct trials (cf. Dutilh et al., 2012) and due to this constraint, only 14,1% of all error trials could be used for plotting. The figure reveals that (i) RTs of errors are significantly slower than RTs of correct responses, (ii) pre-RTs are relatively stable and a pre-error speeding is nearly absent, and (iii) post-error RTs (E + 1) are slower than pre-error RTs (E-1) and this is similar for both error types. Due to the non-representative subset of error trials that could be used for this figure, no further analyses were performed on the depicted pre-error trials even though there appears to be a descriptive difference in RTs before detected and undetected errors.

A paired-sample t-test showed that PES was significantly larger (1476 ± 137 ms) compared to post-correct slowing [PCS; 1446 ± 126 ms; t(20) = 2.151, *p* < .05, d = 0.469]. We then investigated whether PES differed between detected and undetected errors. Differentiating errors into detected and undetected errors did, however, not result in significant differences in PES [PES detected errors = 1465 ± 144 ms, PES undetected errors = 1485 ± 158 ms; t(20) = − 0.826, *p* = .42, d = − 0.180]. Figure 5 illustrates the sequence of RTs surrounding error trials, which reveals that in the SIT, pre-error speeding is nearly absent, and the post-error slowing is similar for detected and undetected errors. In contrast to the PES calculations, the error surrounding trials in the figure are only correct responses (cf.^40^.

Then, we inspected the post-error accuracy (PEA) and found similar effects as for PES. While we did observe a significant difference in PEA (71.3 ± 6.0%) compared to post-correct accuracy [PCA, 74.4 ± 4.2%; t(20) = -2.854, *p* < .01, d = − 0.584], PEA again did not significantly differ between detected and undetected errors [PEA of detected errors: 72.6 ± 7.0%; PEA of undetected errors: 68.8 ± 9.0%, t(20) = 1.770, *p* = .09, d = 0.386]. Thus, also in terms of PEA, the awareness of an error did not influence behavioural adjustments (despite a small trend indicating that PEA might be larger after detected than undetected errors). For information about the development of PES and PEA across time, please see Supplementary Material 3c.

### Discussion study 1

First and foremost, we succeeded in creating an experimental task that produces a large number of undetected errors (more than six errors per error type per person) in an efficient way (i.e., in a short amount of time). The overall error rate was stable across the experiment, which was anticipated given that the adaptive algorithm was adjusted based on the past error rate. Participants generally improved their performance, but this improvement was grounded in RT decreases and avoidance of timeouts – and thus not at the expense of a reduced error rate. This suggests that using the past error rate for adjusting the adaptive algorithm is a promising approach. Moreover, the task also resulted in a stable error detection rate. Importantly, this feature of the current task could be valuable, because an equal distribution of detected and undetected errors throughout the experiment might reduce potential systematic biases (e.g., habituation effects at the beginning of the task or fatigue effects at the end of the task).

Further, the task comprised two hierarchical rules, which had to be applied in 50% of the trials. This reduced surprise effects through infrequent events used in many standard tasks such as Go/Nogo or Flanker tasks. It can be argued that as these two rules had to be memorized, the mnemonic load could negatively impact performance. However, this was the only information that had to be memorized, as stimuli and targets were constantly shown on the screen. In addition, with the extensive training before the start of experiments, it can be assumed that the mnemonic requirements of the task were rather low. While errors occurred similarly often, the errors due to rules 1 and 2 differed in terms of RT pattern and error awareness. While most errors due to rule 2 were detected, errors due to rule 1 were only detected in approximately 50%, i.e., at chance level. We hypothesised that this difference in error detection might have had differential impacts (e.g., better error awareness leads to a reduction in error occurrence). However, this hypothesis was not supported as the error rate and the error detection rate for both rules did not show a differential course throughout the experiment (see Fig. 3A). The reasons underlying the increased number of undetected errors due to rule 1 are going to be further explored in study 2.

Finally, behavioural adjustments in terms of PES and PEA and its relationship with error awareness were explored. As expected, we found a slowing of RTs after errors (i.e., larger PES than PCS), potentially representing adaptive, cautious responding after errors^40^. This pattern, however, was not mirrored in the accuracy measures. We did not observe a better accuracy after errors, which would have been expected due to adaptive, slower responses. Reasons for this observation will be discussed in the general discussion. Interestingly, we could not find an influence of error awareness on any of these behavioural adjustments. With the high statistical power concerning the comparison of detected and undetected errors, we cannot support the claim that error awareness leads to stronger behavioural adjustments^8^. If at all, results showed a small, non-significant effect of larger PEA in the case of detected errors, which is in line with previous accounts^7,8^ but likewise in contrast to others^21,41^. The potential association between behavioural adjustments and error awareness is an important topic in cognitive control research that should be addressed in future studies.

### Study 2

**Objective.** The SIT uses two hierarchical rules, of which one has to be applied on each trial. The first rule implies that a match between stimuli and targets has to be searched (i.e., same category and colour). If no match is present in a given trial, the second rule should be used and implies that the searched target is not represented at all in the stimuli. Study 1 revealed that errors occurring on trials which demanded the application of either of the two rules differed substantially from each other. Importantly, when errors were separated based on the two rules, different levels of error detection were observed (higher detection rates for errors on rule 2 compared to rule 1 errors). The second study was set to explore whether this feature of the SIT could be used to specifically manipulate error detection performance.

**Methods - Specifics of Study 2** While in Study 1, the rules occurred equally often, the frequency of the rules was systematically varied in Study 2. Fifteen experimental blocks with 32 trials were presented (i.e., 480). In the first five blocks, both rules were again equally distributed (50% each rule, “baseline”). For the following five blocks, 75% of the trials demanded applying rule 2 and 25% of the trials the application of rule 1. Finally, this distribution was reversed for the final five blocks (75% rule 1, 25% rule 2). A constant order for every participant was used as piloting work revealed lower error rates when most trials demanded rule 1 (i.e., here, part 3). Hence, the low error rate in these blocks would have resulted in a very low temporal threshold for the timeout. Thus, participants would have been forced to react quickly in these blocks with 75% application of rule 1. If these blocks (with 75% application of rule 1) had preceded the (more challenging) blocks, in which most of the trials demanded rule 2 (i.e., here, part 2), many timeouts would have occurred due to the low temporal threshold based on the previous part. However, we achieved a reasonable temporal threshold in all three parts by keeping the order fixed. In addition, we expanded the output file of the SIT for Study 2 so that more detailed analyses of single trials could be conducted (i.e., outputs included colour and category information about the presented stimuli on each trial).

**Data and statistical analysis.** As a first step, we compared the response rates of the second study with response rates from the first study to test whether the observed behavioural pattern was replicable (especially for the frequency of detected and undetected errors). For that, we averaged the correct rate, error rate, timeout rate, and error detection rate, as well as RTs for correct and errors for the first five blocks of each experiment. In these blocks, the rule frequency was 50:50 in both studies, and thus the comparison was based on the same number of trials collected under similar circumstances (e.g., time on task, habituation, fatigue). The four response rates and RT measures were statistically compared with Welch two-sample independent t-tests.

The next step was to examine whether manipulating the rule frequency led to changes in performance compared to the 50:50 rule distribution. Therefore, we used the first five blocks of the second study as a baseline condition (containing the original 50% rule frequency) and contrasted the response rates of the baseline to the two manipulation conditions. The response rates were averaged for the three parts [i.e., block 1–5 (= baseline, part 1), block 6–10 (part 2) and block 11–15 (part 3)] to evaluate the influence of rule frequency. Paired-sample t-tests were carried out between the baseline and part 2 and the baseline and part 3 for either correct rate, error rate, timeout rate or detection rate.

### Results study 2

For brevity, we only report the results of response rates and RTs here, but please see Supplementary Material 5 for additional results (e.g., behavioural adjustments), which demonstrate further replications of the behavioural patterns observed in study 1. Please note that we refer to Table 1 for all descriptive values corresponding to the below analyses. For visual comparison, the course of response rates and RTs across all blocks can be found in Figs. 2B, 3B and 4B, respectively.

**Descriptive results (averaged across the three parts).** Averaged across all blocks of study 2, the mean error rate was 15.48 ± 6.06% with an error detection rate of 68.37 ± 15.48% (for correct and timeout rates, see Table 1). Thus, on average, 23 undetected errors (range 6–59) per participant were available for statistical analysis. Independent of the manipulation of rule frequency, errors again occurred similarly often for both rules [46.0 ± 13.1% errors due to rule 1; 54.0 ± 13.1% errors due to rule 2; t(19) = -1.371, *p* = .186, d = − 0.307]. Importantly, we also observed a comparable behavioural pattern as in study 1 considering the error detection rate for both rules: While the majority of errors due to the second rule were detected (87.4 ± 16.2%), the detection of errors due to rule 1 was at chance level (48.0 ± 21.2%) [t(19) = -8.042, *p* < .001, d = -1.80]. Lastly, considering RTs, results from study 2 were similar to results from study 1 in the sense that correct responses (1394.5 ± 50.1 ms) had faster RT compared to errors [1726.9 ± 61.1 ms; t(19) = 13.323, *p* < .001, d = 2.98]. To summarize, the overall/ averaged behavioural pattern regarding response rates and RTs was comparable between the two studies. However, the comparison of performance between the three parts was most relevant for study 2.

**Study 2 – Part 1 (Replication).** Overall, the behaviour pattern obtained in study 2 was very similar to the results obtained in study 1, and no significant group differences could be found for the four response types [correct rate t(30.5) = -1.834, *p* > .071, d = − 0.186; error rate t(38.6) = 0.954, *p* > .345, d = 0.147; timeout rate t(38.8) = 0.450, *p* > .655, d = 0.092; error detection rate t(34.1) = 0.288, *p* > .792, d = 0.038]. Thus, the performance and error detection at the beginning of the two studies were similar.

**Fig. 6:**
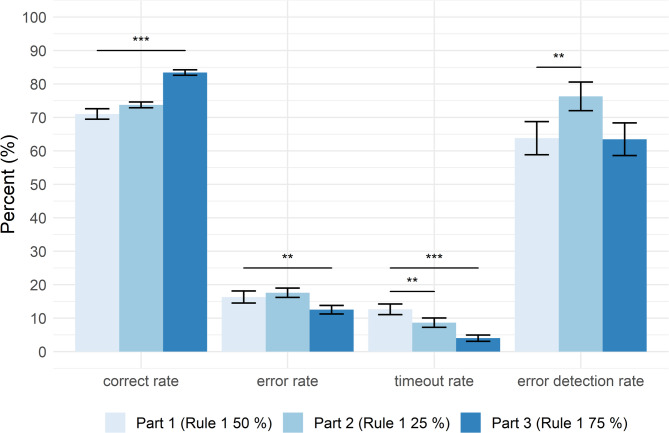
Mean ± standard error of response rates for the three parts of study 2. The correct rate, error rate, and timeout rate are represented as the percentage of all trials, whereas the error detection rate is the percentage of all error trials. Asterisks indicate significant differences in comparison to part 1 (the baseline condition): * *p* < .05; ** *p* < .01; *** *p* < .001.

**Study 2 - Part 2 (Rule 1 applied in 25% of trials).** The paired-sample t-tests contrasting part 2 with the baseline revealed no significant differences in correct and the error rate [t(19) = -1.410, *p* > .174, d = − 0.315 and t(19) = -1.236, *p* > .231, d = − 0.276, respectively]. The rate for timeouts was significantly lower in part 2 than at baseline [t(19) = 3.214, *p* < .005, d = 0.719]. This reduction in the timeout rate might be due to habituation to the task requirements. More importantly, though, and in line with our expectations, we found a significant difference in the error detection rate between part 2 and the baseline [t(19) = -3.330, *p* < .004, d = − 0.745]. Thus, while the correct and error rate were unchanged by the changes in rule frequency, decreasing the number of trials demanding rule 1 led to a specific increase in error detection (see Fig. 6).

**Study 2 - Part 3 (Rule 1 applied in 75% of trials).** In contrast, when adjusting rule frequency in a way that rule 1 should be applied in most trials, we observed the opposite pattern of effects. Compared to the baseline, the correct rate significantly increased [t(19) = -7.562, *p* < .001, d = -1.69], the error rate significantly decreased [t(19) = 2.840, *p* < .010, d = 0.635], and the timeout rate was also significantly lower [t(19) = 6.507, *p* < .001, d = 1.45]. Crucially, the error detection rate was not affected and thus not significantly different compared to the baseline [t(19) = 0.062, *p* > .951, d = 0.014] (see Fig. 6).

### Error sources

Due to the more differentiated information stored in the updated version of the SIT used in study 2, a more detailed analysis of error sources was possible. During the practice sessions before the experiment, participants were trained to apply the two rules hierarchically (i.e., rule 2 can only apply if rule 1 is not applicable). However, there was no explicit cue about which rule should be used within a trial. In the second study, the distribution of errors for the two rules was again similar, replicating results from study 1 despite differences in rule frequency (note that over the whole experiment, independent of the different distributions in parts 2 and 3, both rules again occurred 50% of trials).

At a closer look at potential stimuli for rule 1, it was noticeable that there is always one target whose features are not represented at all in the stimuli - which is the decision criteria for correct targets on trials demanding rule 2 (see, for example, the blue ball in Fig. 1B top). This, of course, only holds if the fact that the rules are hierarchical is ignored. Thus, we wanted to assess how often participants made errors by applying the wrong rule, i.e., rule 2 although rule 1 should have been applied. It turned out that 76.0 ± 10.9% of errors at rule 1 were due to the inappropriate but eventually “correct” application of rule 2. This also explains many undetected errors when rule 2 should have been applied since only a small fraction of these errors was detected by the participants (36.1 ± 23.6%).

### Discussion study 2

The first part of study 2 (baseline) had an identical task design as study 1 and the resulting behavioural pattern for this part of study 2 (RTs of correct and error responses, response rates, behavioural adjustments) was similar to that of study 1. This supports the efficacy of the SIT in producing many (undetected) errors in two independent samples.

The main aim of study 2, however, was to test whether a manipulation of the error detection rate was possible by implementing different frequencies of the rule type. In study 1, we observed that errors got less often detected in trials where rule 1 should have been applied. Thus, we expected that by systematically increasing or decreasing the number of trials applying rule 1, we might also specifically modulate the error detection rate while keeping the error rate unchanged (because there was no difference in error rate between the two rules in study 1). Results of study 2 supported our hypotheses: We could show that error detection was significantly modulated, while at the same time, the error rate remained unchanged, underlining the specificity of the manipulation. To demonstrate the effects of the manipulation, we compared behavioural changes induced by changing rule frequency in comparison to a baseline (i.e., study 2 - part 1, which was identical to the design of study 1, namely a 50:50 rule distribution). The above-described successful modulations occurred in part 2, where we increased the frequency of rule 2.

In contrast, increasing the frequency of rule 1 (i.e., part 3 compared to the baseline) resulted in increased correct responses and decreased errors but did not affect the error detection rate. The SIT was initially developed to provoke many undetected errors, but as shown in study 2, the SIT can easily be adjusted to produce variations of specific outcome variables and, for instance, provoke fewer undetected errors (if this outcome pattern is desired). Another possibility to explain these results are related to task habituation or learning effects, which were possible because of the implementation of a fixed sequence of the parts. Speaking against this argumentation, however, is the fact there is no gradual decrease in the error rate and the increase in error detection vanished again in part 3. Independent of that, we observed that avoiding timeouts seemed to reflect general learning processes throughout the task.

Finally, study 2 led to further differentiation of error types. While in study 1, it was apparent that errors due to rules 1 and 2 were fundamentally different, we further identified sources leading to (mostly undetected) errors within the category of rule 1 errors: Rule 1 errors could be differentiated based on whether rule 2 was “correctly” applied or not, i.e., whether the error was due to a misclassification about which rule should have been applied. Depending on whether or not rule 2 was applied to errors due to rule 1, the error types differed in terms of awareness and RTs (see Supplementary Material 5 for RT results). Knowing about the different error types and possibilities of provoking or countervailing them is a valuable benefit of the presented task.

### General discussion

We here presented a new paradigm for assessing error awareness. The Speeded Inference Task (SIT) overcomes several weaknesses of past attempts with the main goal to provoke many undetected errors. In study 1, we succeeded in generating a high, stable error rate with many undetected errors (more than six errors per errors type per participant). The stability of the error rate suggests that errors did not primarily occur due to fatigue, but rather due to cognitive conflict as intended by us. Another finding supporting this claim relates to the observed longer RTs for errors than correct responses. While studies on cognitive control processes commonly report faster RTs for errors and slower RTs for correct responses, recent studies implementing more cognitively challenging tasks reported the opposite pattern. This discrepancy likely reflects differences in the underlying causes of errors. In relatively simple tasks, such as two-choice flanker tasks, errors often result from fast, premature response reflecting a failure of motor inhibition^39^. In contrast, in more challenging tasks, errors could occur due to lapses in memory, weakness in attention or deficits in evidence accumulation, which all might delay responses (i.e., RTs close to the response deadline;^42–46^). Hence, when error RTs are prolonged as in our studies, this pattern speaks in favour of cognitive processing deficits, rather than premature responding, underlying the observed errors.

In study 1, we further assessed behavioural adjustments in terms of PES and PEA as well as its relationship with error awareness. First, we found a slowing of RTs after errors (i.e., larger PES than PCS), but unexpectedly no better accuracy after errors. While this might seem counterintuitive at first sight, this finding can be explained by the increased frequency of timeouts following errors. Due to the (relatively) short response deadline, a stronger slowing of responses after errors leads to more occurrences of timeouts – which likewise leads to reduced post-error accuracy. Thus, the initial behavioural adjustments in RT, therefore, might be adaptive, but due to the specific features of the task (e.g., the changing, unpredictable temporal threshold of timeouts) eventually result in worse performance in terms of post-error accuracy. On the other hand, we observed clear adaptive adjustments in terms of speeding RTs after timeouts (details in Supplementary Material 3)^12^. The source for a timeout is obviously a too-slow response; hence to counteract these concrete error sources, the performance monitoring system speeds up following responses.

Furthermore, there was no apparent relationship between error awareness and PES or PEA, except a non-significant trend indicating that PEA might be larger after detected than undetected errors. If this were true, the current findings would support the idea that error awareness and error processing are two independent processes in a more general sense (e.g., automatic adjustments to errors)^2,47^. However, the results of behavioural adjustments have to be treated with caution. Even though error awareness is commonly assessed by asking participants to evaluate their performance on every trial^6,21,48^, this draws their attention towards error commission and might disrupt post-error processes^12,49^. Thus, behavioural adjustments measured in the SIT are not comparable to adjustments in everyday life situations and in tasks without awareness rating. However, the SIT was created with a focus on error awareness (rather than post-error processing), and the difficult endeavour of establishing a better way to reliably measure the relationship between error awareness and behavioural adjustments (cf.^41^. could be tackled in future studies.

A final interesting observation from study 1 was related to the occurrence of timeouts. The timeouts were initially included in the SIT to induce time pressure and to implement the adaptive algorithm for adjusting the task difficulty level. Timeouts occurred on average in 8% of all trials and are, by definition, too-slow responses and, therefore, incorrect events. While the usage of timeouts also has disadvantages (e.g., loss of post-error response times in case of timeouts in trials after errors), it is a crucial feature of the current task^22^ to guarantee a high and stable error rate. Also, it allowed a thorough analysis of behavioural adjustments in response to timeouts: There was a descriptive difference in the magnitude of the RT adjustments, as they were twice as large for timeouts (post-timeout speeding) compared to the RT changes after errors (post-error slowing). The difference between the two conditions was that errors had to be detected by the participants, while timeouts resulted in full awareness of unsuccessful trials, since the correct answer was explicitly shown. Thus, despite the above-described missing relationship between error awareness and PES/ PEA, the current results on timeouts might suggest that there could be a relationship (full awareness leading to large adjustment) that yet has to be fully explored.

Study 2 generated a comparable behavioural pattern to study 1 by using the same design in a different sample (related to part 1 of study 2), thus replicating the positive outcome of study 1. In addition, study 2 proved that specific features of the task are relevant for error awareness and that changing these features (here rule frequency) allows to modulate awareness in a within-subject manner. Interestingly, we observed that error detection changed independently of the accuracy in part 2, while in part 3 the reverse pattern was observed, i.e., accuracy changed, and the error detection rate remained unchanged. As discussed above, these results again hint towards (at least in part) separate mechanisms supporting error processing and error awareness: While error processing (including behavioural adjustments) might be associated with processes on a more global level, eventually resulting in the accuracy of a person, the (error) awareness might be related to meta-cognition and/ or processes on a higher cognitive level^2,47,50^. Therefore, it seems that manipulation of one measure (error rate/accuracy, error awareness) is possible without affecting the other.

### Limitations and future directions

As we introduced a new paradigm, we observed benefits and disadvantages of this new approach. Therefore, we discuss the limitations of the SIT here, so that these can be addressed. For instance, we created a paradigm that should be challenging and, at the same time, not relying on too many mnemonic aspects. Therefore, it was necessary to increase the complexity at several stages (e.g., four possible response buttons, implementation of category-colour matches, and hierarchical rules). To counteract the complexity, we used comprehensive practice sessions before the start of the main task to ensure an internalised category-colour matching.

After the experiments, participants anecdotally reported that they felt very motivated throughout the task to perform well, especially towards the end of the task, which can be attributed to the invented scoring system. While this was one of the goals for our new paradigm, the motivation of participants should be systematically assessed in future studies. Besides, the rewarding scheme was intended to reward correct responses, but future studies could explore whether an exponential rewarding scheme, i.e., rewarding faster correct RTs more than slower correct RTs, is even more effective in motivating participants and/or may result in lower/ diminishing timeout rates.

Furthermore, we suggest using only two options for the response evaluation for future studies, because the two ‘maybe’ options were rarely chosen. Depending on the population of interest, it might be beneficial to include options to express uncertainty of the evaluation^22,48^, but for a healthy, young sample, a binary response evaluation might be sufficient. That the participants were quite confident in their response evaluation indicated that they properly understood (and performed) the task and that they were generally able to detect their errors. The constant and high number of detected and undetected errors might allow the application of computational modelling in future research. In this case, two-choice rating for the response evaluation would be necessary and researcher could apply drift diffusion models (DDM) such as the Bayesian hierarchical DDM, to better understand the processes underlying error awareness 

Lastly, considering the analysis, there are three points we want to stress. First, as mentioned before, the effect sizes and hence sample size could not be estimated a priori for this newly developed task. We included a relatively small sample, because the majority of goals were only descriptive in nature, and this sample size provided 80% power to detect effects of d ≥ 0.635. Yet it is possible that the two studies were underpowered. Future studies should replicate our findings (especially those of the rule frequency manipulation in study 2) to back up the here reported effect sizes. Second, as we saw in studies 1 and 2, errors for the two rules differ in their error detection rate. Therefore, the differences between the two rules might be confounded by the underlying differences in error awareness. Third, the utility of the timeouts needs to be reassessed. While they are helpful for the implementation of the adaptive response deadline, timeout trials also introduce a new and separate response type. Participants did not respond in time, but it remains unknown at which stage in the participants’ decision-making process the timeout occurred. Therefore, the processes leading to and the effects of timeouts need further investigation.

As mentioned above, the paradigm was designed flexibly, allowing easy modifications depending on the research question. In study 2, we proved that rule frequency changes could modulate error detection. The task combines several features planned to make the layout appealing to participants (in contrast to abstract symbols often used in conflict task, like the Flanker tasks). Whether or not participants in these 2 presented studies were more motivated than when conducting standard conflict tasks warrants further exploration.

Within the current studies, we aimed to achieve an error rate of approximately 20%, but the adaptive algorithm can easily be changed for future studies with higher or lower aspired error rates. Note that the chosen adaptive algorithm (i.e., ± 10% of the current response threshold) was based on piloting work intended to produce stable error rates (which it successfully achieved). Depending on the research question and the specific goal of the algorithm, other implementations (e.g., based on cumulative RT distribution) are conceivable and should be further explored. Whether it is feasible to use the SIT without an adaptive algorithm at all remains another open question and depends on the specific research question. Having an experimental task that adjusts its difficulty level to the participants’ performance will most likely result in the adoption of different cognitively controlled strategies in contrast to tasks without the dynamic adjustments. Therefore, the results obtained from the SIT cannot readily be compared to results from former studies on error awareness that did not implement an adaptive algorithm. Finally, because the stimuli were shown on the screen until a response was made, we could ensure that errors did not occur due to perceptual difficulties but were more likely to be related to higher-order cognitive processes (e.g., as described above for the identified rule application errors).

To conclude, we introduced a new behavioural paradigm suitable for assessing error awareness. It provoked many undetected errors and a stable error rate across time in young adults, which has been a challenging endeavour^21–23^. We identified different types of errors, which could be further explored with electrophysiological measures to understand better the neural processes underlying error processing and error awareness. Considering behavioural adjustments after errors, our results showed that neither PES nor PEA were (strongly) influenced by error awareness, supporting the idea that control processes after errors and error awareness might rely on (at least in part) separate mechanisms.

## Supplementary Information

Below is the link to the electronic supplementary material.

Supplementary Material 1

## Data Availability

The datasets generated and/or analysed during the current study are not publicly available because we did not explicitly ask participants for consent of publication for their anonymised individual data, but they are available from the corresponding author on reasonable request. The open source code for the application of the Speeded Inference Task (SIT) can be found on github (https://github.com/EvaNiessen/Speeded-Inference-Task).

## Footnotes

1 The experiment was scripted in PsychoPy [38,39], and the open-source code and a short video for illustrations can be found on GitHub (https://github.com/EvaNiessen/Speeded-Inference-Task).

2 Correct detection rate was calculated as correct responses rate das correct divided by the overall number of correct responses.

## References

[1] Nieuwenhuis, S., Ridderinkhof, K. R., Blom, J., Band, G. P. & Kok, A. Error-related brain potentials are differentially related to awareness of response errors. Evidence from an antisaccade task. *Psychophysiology* **38**, 752–760 (2001). 11577898

[2] Di Gregorio, F., Maier, M. E. & Steinhauser, M. Are errors detected before they occur? Early error sensations revealed by metacognitive judgments on the timing of error awareness. *Conscious. Cogn.* **77**, 102857 (2020). 31837572 10.1016/j.concog.2019.102857

[3] O’Connell, R. G. et al. The role of cingulate cortex in the detection of errors with and without awareness: a high-density electrical mapping study. *Eur. J. Neurosci.* **25**, 2571–2579 (2007). 17445253 10.1111/j.1460-9568.2007.05477.x

[4] Botvinick, M. M., Braver, T. S., Barch, D. M., Carter, C. S. & Cohen, J. D. Conflict Monitoring and Cognitive Control. *Psychol. Rev.* **108**, 624–652 (2001). 11488380 10.1037/0033-295x.108.3.624

[5] Holroyd, C. B. & Coles, M. G. H. The neural basis of human error processing: reinforcement learning, dopamine, and the error-related negativity. *Psychol. Rev.* **109**, 679–709 (2002). 12374324 10.1037/0033-295X.109.4.679

[6] Hester, R., Foxe, J. J., Molholm, S., Shpaner, M. & Garavan, H. Neural mechanisms involved in error processing: a comparison of errors made with and without awareness. *NeuroImage* **27**, 602–608 (2005). 16024258 10.1016/j.neuroimage.2005.04.035

[7] Nieuwenhuis, S., Ridderinkhof, K. R., Blom, J., Band, G. P. & Kok, A. Error-related brain potentials are differentially related to awareness of response errors: evidence from an antisaccade task. *Psychophysiology* (2001). 11577898

[8] Klein, T. A. et al. Neural correlates of error awareness. *NeuroImage* **34**, 1774–1781 (2007). 17185003 10.1016/j.neuroimage.2006.11.014

[9] Danielmeier, C. & Ullsperger, M. Post-error adjustments. *Front. Psychol.* **2**, 233 (2011). 21954390 10.3389/fpsyg.2011.00233 PMC3173829

[10] Endrass, T., Reuter, B. & Kathmann, N. ERP correlates of conscious error recognition: aware and unaware errors in an antisaccade task. *Eur. J. Neurosci.* **26**, 1714–1720 (2007). 17880402 10.1111/j.1460-9568.2007.05785.x

[11] Notebaert, W. et al. Post-error slowing: an orienting account. *Cognition* **111**, 275–279 (2009). 19285310 10.1016/j.cognition.2009.02.002

[12] Wessel, J. R. An adaptive orienting theory of error processing. *Psychophysiology* **55** (2018). 10.1111/psyp.13041 29226960

[13] Steele, V. R. et al. Neuroimaging measures of error-processing: Extracting reliable signals from event-related potentials and functional magnetic resonance imaging. *NeuroImage* **132**, 247–260 (2016). 26908319 10.1016/j.neuroimage.2016.02.046 PMC4860744

[14] Pontifex, M. B. et al. On the number of trials necessary for stabilization of error-related brain activity across the life span. *Psychophysiology* **47**, 767–773 (2010). 20230502 10.1111/j.1469-8986.2010.00974.x

[15] Schreiber, M., Endrass, T., Weigand, A. & Kathmann, N. Age Effects on Adjustments of Performance Monitoring to Task Difficulty. *J. Psychophysiol.* **26**, 145–153 (2012).

[16] Somon, B., Campagne, A., Delorme, A. & Berberian, B. Evaluation of performance monitoring ERPs through difficulty manipulation in a response-feedback paradigm. *Brain Res.* **1704**, 196–206 (2019). 30300637 10.1016/j.brainres.2018.10.007

[17] Boksem, M. A. S., Meijman, T. F. & Lorist, M. M. Mental fatigue, motivation and action monitoring. *Biol. Psychol.* **72**, 123–132 (2006). 16288951 10.1016/j.biopsycho.2005.08.007

[18] Xiao, Y. et al. Sustained attention is associated with error processing impairment: evidence from mental fatigue study in four-choice reaction time task. *PloS one*. **10**, e0117837 (2015). 25756780 10.1371/journal.pone.0117837 PMC4355415

[19] Bode, S. & Stahl, J. Predicting errors from patterns of event-related potentials preceding an overt response. *Biol. Psychol.* **103**, 357–369 (2014). 25450163 10.1016/j.biopsycho.2014.10.002

[20] Mattes, A., Porth, E. & Stahl, J. Linking neurophysiological processes of action monitoring to post-response speed-accuracy adjustments in a neuro-cognitive diffusion model. *NeuroImage* **247**, 118798 (2022). 34896290 10.1016/j.neuroimage.2021.118798

[21] Kirschner, H., Humann, J., Derrfuss, J., Danielmeier, C. & Ullsperger, M. Neural and behavioral traces of error awareness. *Cogn. Affect. Behav. Neurosci.* **21**, 573–591 (2021). 33025512 10.3758/s13415-020-00838-w PMC8208913

[22] Niessen, E., Fink, G. R., Hoffmann, H. E. M., Weiss, P. H. & Stahl, J. Error detection across the adult lifespan: Electrophysiological evidence for age-related deficits. *NeuroImage* **152**, 517–529 (2017). 28284803 10.1016/j.neuroimage.2017.03.015

[23] Rabbitt, P. Consciousness is slower than you think. *The Quaterly J. Experimental Psychology*, 1081–1092 (2002). 10.1080/02724980244000080 12420985

[24] Cohen, M. X., van Gaal, S., Ridderinkhof, K. R. & Lamme, V. A. F. Unconscious errors enhance prefrontal-occipital oscillatory synchrony. *Front. Hum. Neurosci.* **3**, 54 (2009). 19956401 10.3389/neuro.09.054.2009 PMC2786300

[25] Steinhauser, M. & Yeung, N. Decision processes in human performance monitoring. *J. Neurosci.* **30**, 15643–15653 (2010). 21084620 10.1523/JNEUROSCI.1899-10.2010 PMC3073548

[26] Endrass, T., Klawohn, J., Preuss, J. & Kathmann, N. Temporospatial dissociation of Pe subcomponents for perceived and unperceived errors. *Front. Hum. Neurosci.* **6**, 178 (2012). 22737113 10.3389/fnhum.2012.00178 PMC3381446

[27] Harty, S. et al. Transcranial direct current stimulation over right dorsolateral prefrontal cortex enhances error awareness in older age. *J. neuroscience: official J. Soc. Neurosci.* **34**, 3646–3652 (2014). 10.1523/JNEUROSCI.5308-13.2014 PMC6608991 24599463

[28] Steinhauser, M. & Yeung, N. Error awareness as evidence accumulation. Effects of speed-accuracy trade-off on error signaling. *Front. Hum. Neurosci.* **6**, 240 (2012). 22905027 10.3389/fnhum.2012.00240 PMC3417303

[29] Steinhauser, M. & Yeung, N. Error awareness as evidence accumulation: effects of speed-accuracy trade-off on error signaling. *Front. Hum. Neurosci.* **6**, 240 (2012). 22905027 10.3389/fnhum.2012.00240 PMC3417303

[30] Murphy, P. R., Robertson, I. H. & Harty, S. & O’Connell, R. G. Neural evidence accumulation persists after choice to inform metacognitive judgments. *eLife* 4 (2015). 10.7554/eLife.11946 PMC4749550 26687008

[31] Harsay, H. A. et al. Error blindness and motivational significance: Shifts in networks centering on anterior insula co-vary with error awareness and pupil dilation. *Behav. Brain. Res.* **355**, 24–35 (2018). 29107022 10.1016/j.bbr.2017.10.030

[32] Di Rosa, E., Masina, F., Vallesi, A. & Mapelli, D. The Role of Motivation and Anxiety on Error Awareness in Younger and Older Adults. *Front. Psychiatry*. **12**, 567718 (2021). 33679465 10.3389/fpsyt.2021.567718 PMC7933585

[33] Hajcak, G., Moser, J. S., Yeung, N. & Simons, R. F. On the ERN and the significance of errors. *Psychophysiology* **42**, 151–160 (2005). 15787852 10.1111/j.1469-8986.2005.00270.x

[34] Dignath, D., Eder, A. B., Steinhauser, M. & Kiesel, A. Conflict monitoring and the affective-signaling hypothesis-An integrative review. *Psychon. Bull. Rev.* **27**, 193–216 (2020). 31898269 10.3758/s13423-019-01668-9

[35] Lumsden, J., Edwards, E. A., Lawrence, N. S., Coyle, D. & Munafò, M. R. Gamification of Cognitive Assessment and Cognitive Training: A Systematic Review of Applications and Efficacy. *JMIR serious games* 4, e11 (2016). 10.2196/games.5888 PMC4967181 27421244

[36] Friehs, M. A., Dechant, M., Vedress, S., Frings, C. & Mandryk, R. L. Effective Gamification of the Stop-Signal Task: Two Controlled Laboratory Experiments. *JMIR serious games*. **8**, e17810 (2020). 32897233 10.2196/17810 PMC7509611

[37] Oldfield, R. C. The assessment and analysis of handedness: The Edinburgh inventory. *Neuropsychologia*, 97–113 (1971). 10.1016/0028-3932(71)90067-4 5146491

[38] Rabbitt, P. M. Errors and error correction in choice-response tasks. *J. Exp. Psychol.* **71**, 264–272 (1966). 5948188 10.1037/h0022853

[39] Stahl, J. et al. Neural correlates of error detection during complex response selection: Introduction of a novel eight-alternative response task. *Biol. Psychol.* **156**, 107969 (2020). 33058968 10.1016/j.biopsycho.2020.107969

[40] Ridderinkhof, K. R. Micro- and macro-adjustments of task set: activation and suppression in conflict tasks. *Psychol. Res.* **66**, 312–323 (2002). 12466928 10.1007/s00426-002-0104-7

[41] Dali, G., Orr, C. & Hester, R. Error awareness and post-error slowing: The effect of manipulating trial intervals. *Conscious. Cogn.* **98**, 103282 (2022). 35085977 10.1016/j.concog.2022.103282

[42] Shalgi, S., O’Connell, R. G., Deouell, L. Y. & Robertson, I. H. Absent minded but accurate: delaying responses increases accuracy but decreases error awareness. *Exp. Brain Res.* **182**, 119–124 (2007). 17634930 10.1007/s00221-007-1054-5

[43] Overhoff, H. et al. The relationship between response dynamics and the formation of confidence varies across the lifespan. *Front. Aging Neurosci.* **14**, 969074 (2022). 36589534 10.3389/fnagi.2022.969074 PMC9799236

[44] Keuleers, E., Diependaele, K. & Brysbaert, M. Practice effects in large-scale visual word recognition studies: a lexical decision study on 14,000 dutch mono- and disyllabic words and nonwords. *Front Psychol* **1**, 174 . 10.3389/fpsyg.2010.00174 PMC3153785 21833236

[45] Alós-Ferrer, C. & Garagnani, M. Errors, fast and slow. *Cogn. Psychol.* **162**, 101779 (2026). 41389757 10.1016/j.cogpsych.2025.101779

[46] Porth, E., Mattes, A. & Stahl, J. Motor inhibition errors and interference suppression errors differ systematically on neural and behavioural features of response monitoring. *Sci. Rep.* **14**, 15966 (2024). 38987364 10.1038/s41598-024-66364-8 PMC11237018

[47] Charles, L., van Opstal, F., Marti, S. & Dehaene, S. Distinct brain mechanisms for conscious versus subliminal error detection. *NeuroImage* **73**, 80–94 (2013). 23380166 10.1016/j.neuroimage.2013.01.054 PMC5635965

[48] Overhoff, H. et al. Neural correlates of metacognition across the adult lifespan. *Neurobiol. Aging*. **108**, 34–46 (2021). 34487950 10.1016/j.neurobiolaging.2021.08.001

[49] Porth, E., Mattes, A. & Stahl, J. The influence of error detection and error significance on neural and behavioral correlates of error processing in a complex choice task. *Cogn. Affect. Behav. Neurosci.* **22**, 1231–1249 (2022). 35915335 10.3758/s13415-022-01028-6 PMC9622536

[50] Wessel, J. R. Error awareness and the error-related negativity: evaluating the first decade of evidence. *Front. Hum. Neurosci.* **6**, 88 (2012). 22529791 10.3389/fnhum.2012.00088 PMC3328124

[51] Peirce, J. W. PsychoPy–Psychophysics software in Python. *J. Neurosci. Methods*. **162**, 8–13 (2007). 17254636 10.1016/j.jneumeth.2006.11.017 PMC2018741

[52] Peirce, J. W. Generating Stimuli for Neuroscience Using PsychoPy. *Front. neuroinformatics*. **2**, 10 (2008). 10.3389/neuro.11.010.2008 PMC2636899 19198666

[53] Pfister, R. & Foerster, A. How to measure post-error slowing: The case of pre-error speeding. *Behav. Res. Methods*. **54**, 435–443 (2022). 34240334 10.3758/s13428-021-01631-4 PMC8863758

