# Supplementary material for: Characterizing the behavioural correlates of error awareness with the novel speeded inference task
Source: Sci Rep. 2026 Jun 3;16:17137. doi: 10.1038/s41598-026-56114-3 (PMC13234323; doi:10.1038/s41598-026-56114-3)
Supplement: Supplementary file 1 — Supplementary Material 1 [file 41598_2026_56114_MOESM1_ESM.docx]

##### **Characterizing the behavioural correlates of error awareness with the novel Speeded Inference Task**

##### **Niessen, Wickert, Schober, Fink, Weiss & Stahl**

**Supplemental Material**

1. Collection of stimuli

Used pictures in the Speeded Inference Task (SIT), representing four categories (balls, birds, ice creams and chairs). For each category, one picture was used as the target (middle picture), and seven further pictures served as stimuli (surrounding pictures). These were shown to all participants before starting the task.


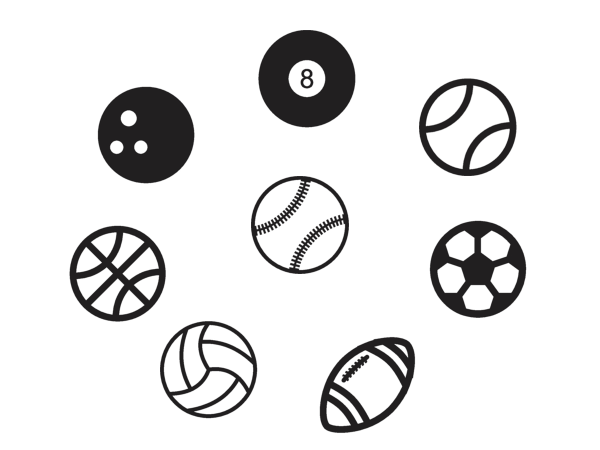

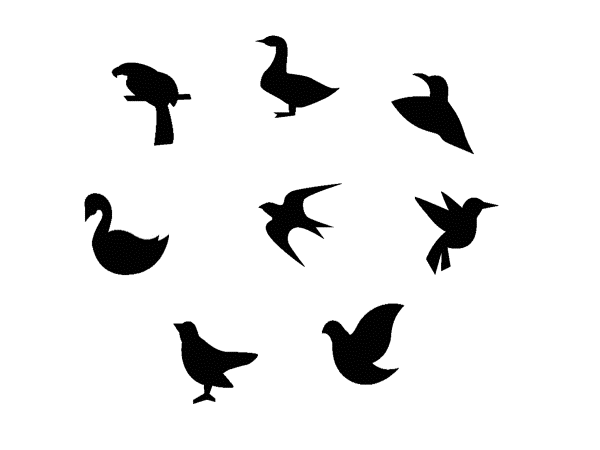

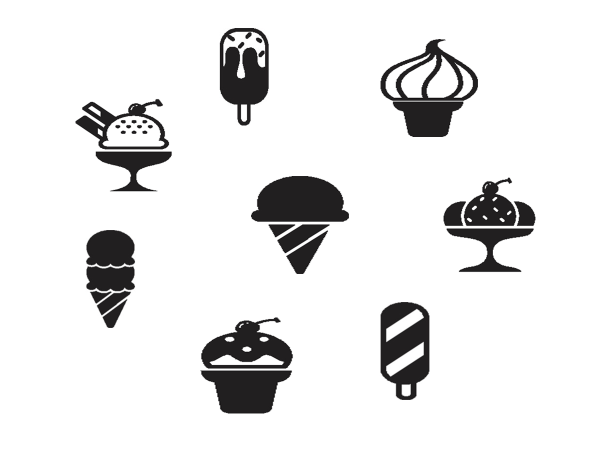

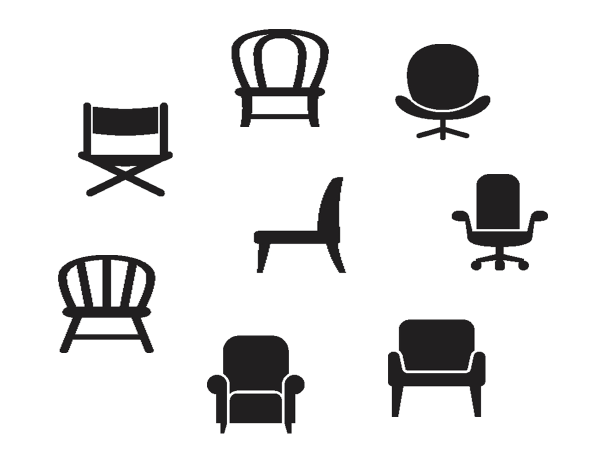


1. Task instructions

Participants read the instructions in a self-paced manner, meaning that there was no temporal limit during this period. The task was introduced together with exemplary stimuli for visualization in separate slides and whenever ready, participants could proceed to the next slide via button press. The English version of the instructions read as follows:

"Your task is to respond as quickly and accurately as possible to shown pictures. Please use the keys under your left and right middle and index finger throughout the whole experiment.

Four categories of targets will constantly be shown: a ball, an ice cream, a chair and a bird. Each target carries one specific colour. Above you can see an example for a category-colour matching.

On each trial, a picture will be shown above the targets that consists of two components: Two examples of the target categories in different colours. These pictures indicate which target in that trial should be chosen.

The pictures are never identical with the targets, important is only the associated category. Thus, every shown bird shall be treated as bird - independent of the way of presentation. Here you see all pictures of birds that we will use.

Here you see all pictures of ice creams that we will use. Take a closer look. (*similar for balls and chairs*).

Two rules decide which target is correct. The first rule is easy: If one of the four targets is identical with one of the shown pictures (in category and colour), then this target should be chosen. In the example: blue ball.

The second rule is more complicated: If none of the four targets is identical to one of the presented pictures above, then you should choose the target that is neither represented in category nor in colour. In the example: green ice cream.

Please note: The time for your response is restricted. If you are too slow, this will be illustrated by the signal \"Timeout\". In case of a timeout, the computer will randomly choose one of the targets.

After one target was chosen (either by you or the computer) you are asked to evaluate the choice. You can use our four-point scale with your four fingers for that.

To reward a good performance, we created a scoring system: For every correctly chosen target, you earn 10 points, and for every correct evaluation of the target, you earn 3 points.

Breaks will be given throughout the experiment. The duration of the breaks is up to you. In these breaks, you can inspect your current score (in comparison to the maximum possible score) as well as a highscore. If you manage to crack our record, a surprise is waiting for you!

If you don't have any more questions, we will first start with a short introduction to the task (with feedback) and a training session (without feedback) before we start the real experiment. Good luck and have fun!"

1. Additional results of study 1
2. *Results concerning the occurrence of timeouts*

**Analysis.** As the occurrence of timeouts was an interesting feature of the SIT, we wished to better understand behavioural changes associated with timeouts. A timeout can be considered a too-slow and incorrect response that participants should avoid. Therefore, besides examining the descriptive properties of timeouts, we computed the RTs after timeouts (post-timeout speeding, PTS) and contrasted those with RTs after correct responses (PCS) - again with a paired-sample t-test. Finally, we hypothesised that prolonged RTs due to a strong PES could likewise result in a greater number of timeouts after errors. To verify this, we computed the frequency of timeouts after errors and contrasted this with the frequency after correct responses.

**Descriptive results.** Most automatically chosen targets after timeouts were incorrect (i.e., 73.7 ± 8.0 %). Considering the ratings of computer-generated responses after timeouts, 79.8 ± 16.2 % had been correctly evaluated by the participants (i.e., correct responses were rated as correct, and errors were rated as incorrect). Finally, most timeouts occurred on trials where rule 2 was applied (85.4 ± 6.2 %).

**Behavioural adjustments to timeouts.** Finally, we examined two measures related to the occurrence of timeouts. First, regarding RT adjustments after timeouts, we observed a significant speeding of RTs after timeouts [-84.9 ± 136.8 ms] compared to PCS [-0.2 ± 21.0 ms; t(20) = -2.613, p = .017, d = -.570]. Second, as expected, the frequency of timeouts was significantly higher after errors (9.6 ± 4.3 %) compared to after correct responses [7.5 ± 3.5 %, t(20) = 2.304, p = .032, d = .503].

1. *Influence of rule type on RTs*

To investigate the potential influence of rule type on RTs, a 2x2 RM ANOVA with the factors rule and accuracy was conducted. Results showed a main effect of rule [F(1,80) = 130.463, p < .001, η2G = .373] with slower RTs for rule 2 (1746.1 ± 174.9 ms) than for rule 1 (1361.4 ± 288.7 ms). As already seen, correct responses (1437.6 ± 366.1 ms) had faster RT compared to errors [1669.9 ± 167.6 ms; F(1,80) = 47.599, p < .001, η2G = .620]. However, the interaction between accuracy and rule was also significant [F(1,80) = 68.514, p < .001, η2G = .461]. Post-hoc tests revealed that RTs were fastest for correct responses on rule 1 (1105.8 ± 107.7 ms), followed by errors on rule 1 (1617.0 ± 148.7 ms) and errors and correct responses on rule 2 (1722.9 ± 172.0 ms; 1769.3 ± 3.3 ms, respectively; all p < .001). This indicates that the main effect of rule type on RT was mainly driven by the difference in RT for correct and incorrect responses in trials in which rule 1 should have been applied.

1. *Behavioural adjustments across block*

To examine a possible change in adjustments across time, a 2x14 RM ANOVA was conducted with the factors accuracy (post-correct and post-error) and block (1-14 blocks) separately for response times and accuracy (thus PES vs. PCS and PEA vs. PCA). For PES, there was neither a main effect of block [F(13,143) = 1.235, p = .260, η2G = .034], nor a main effect of accuracy [F(1,11) = 2.925, p = .115, η2G = .012], as well as no significant interaction [F(13,143) = 1.041, p = .416, η2G = .055]. For a visual illustration, please see Figures below.


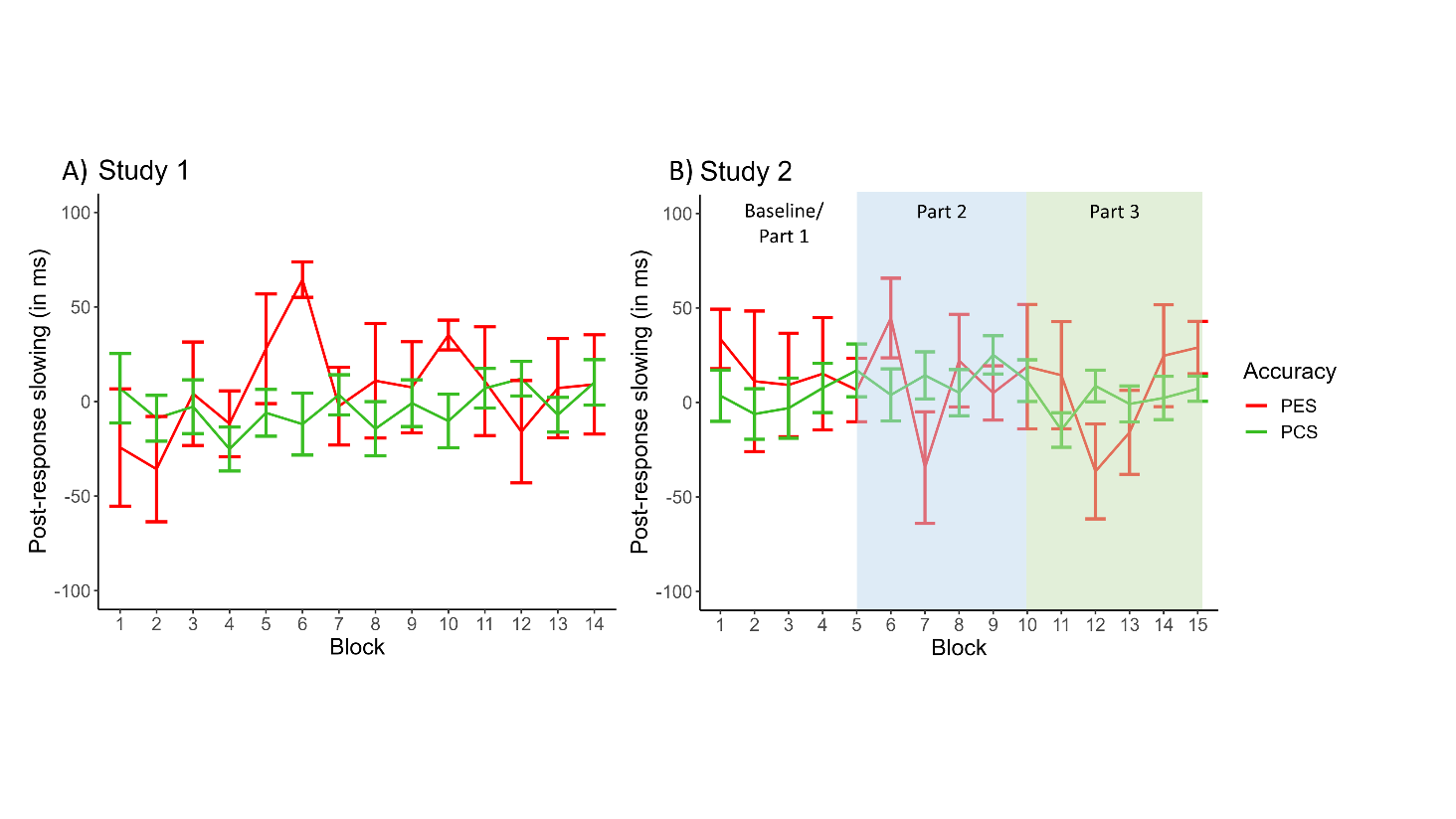


Post-response slowing (difference post-pre trial RT) for post-error trials (red line, PES) and post-correct trials (green line, PCS) are shown across all blocks for study 1 (A) and study 2 (B). Mean ± standard error are plotted.


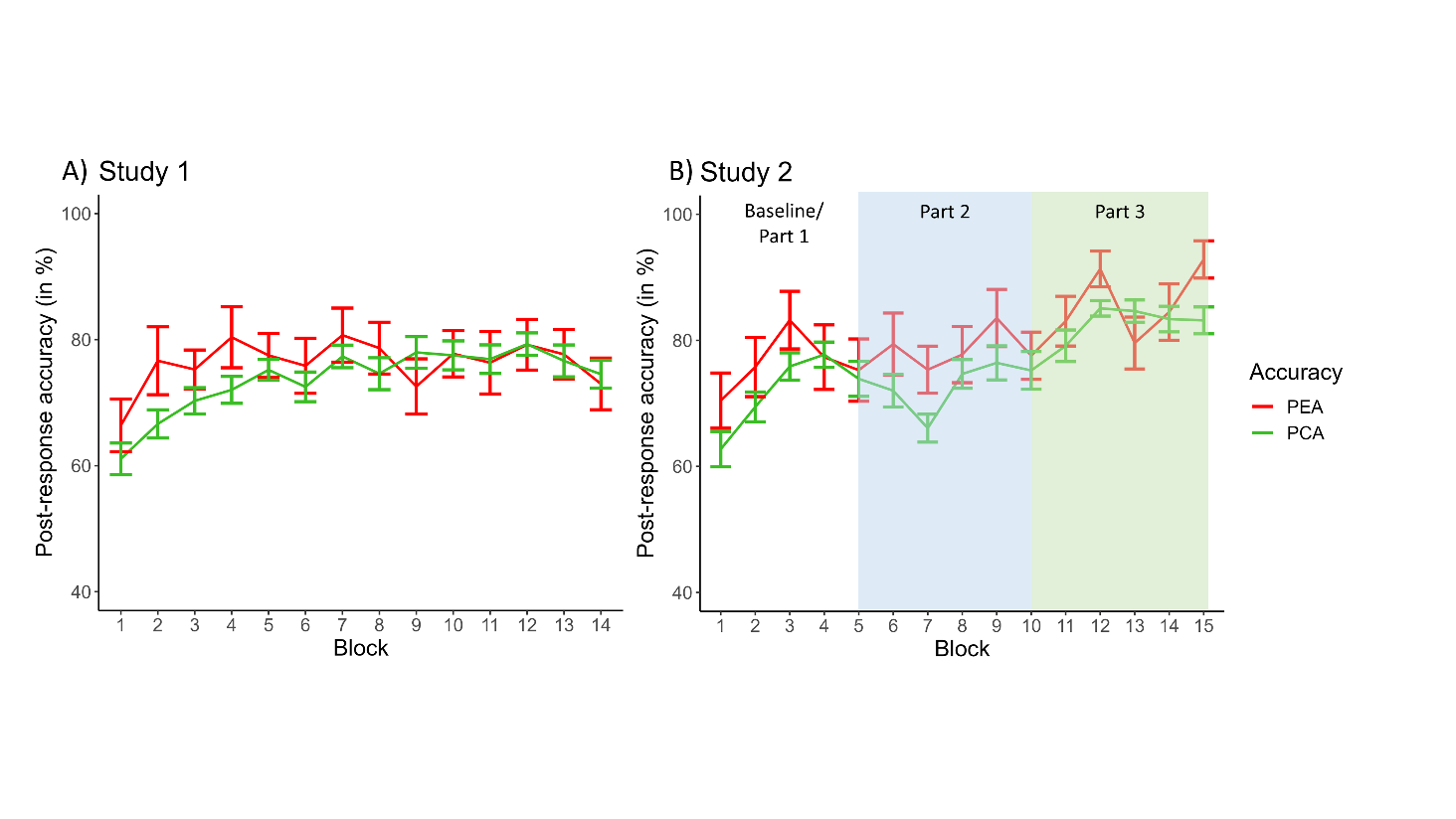


Post-response accuracy (percent correct responses) for post-error trials (red line, PEA) and post-correct trials (green line, PCA) are shown across all blocks for study 1 (A) and study 2 (B). Mean ± standard error are plotted.

1. Individual differences in error likelihood due to the two rules

Even though, on average, errors for both rules occurred similarly often (see main manuscript), we noticed individual differences. In a post-task questionnaire, participants often reported experiencing one rule being more complex than the other. We assumed that underlying strategic differences might be the reason for the individual differences (e.g., focussing on identifying a not-matching stimulus first, despite rule 1 might apply). Implementing deliberate strategies and potential consequences on performance and error awareness should be further explored in studies employing larger samples.

The two plots below show how many percent of all errors were due to rule 1 (lighter red) and rule 2 (darker red) for each participant. The dashed line at 50 % illustrates an equal distribution. Within study 1, nine participants made similar errors due to both rules (40-60%). Nine participants experienced significant difficulties with rule 2; hence, more than 60 % of errors were due to rule 2. Only three participants showed the opposite pattern and made the most errors (>60%) due to rule 1. This grouping of participants according to their error distribution looked similar for participants in study 2 (eight participants had equal errors on both rules, eight participants had more errors due to rule 2, and four participants had more errors due to rule 1).


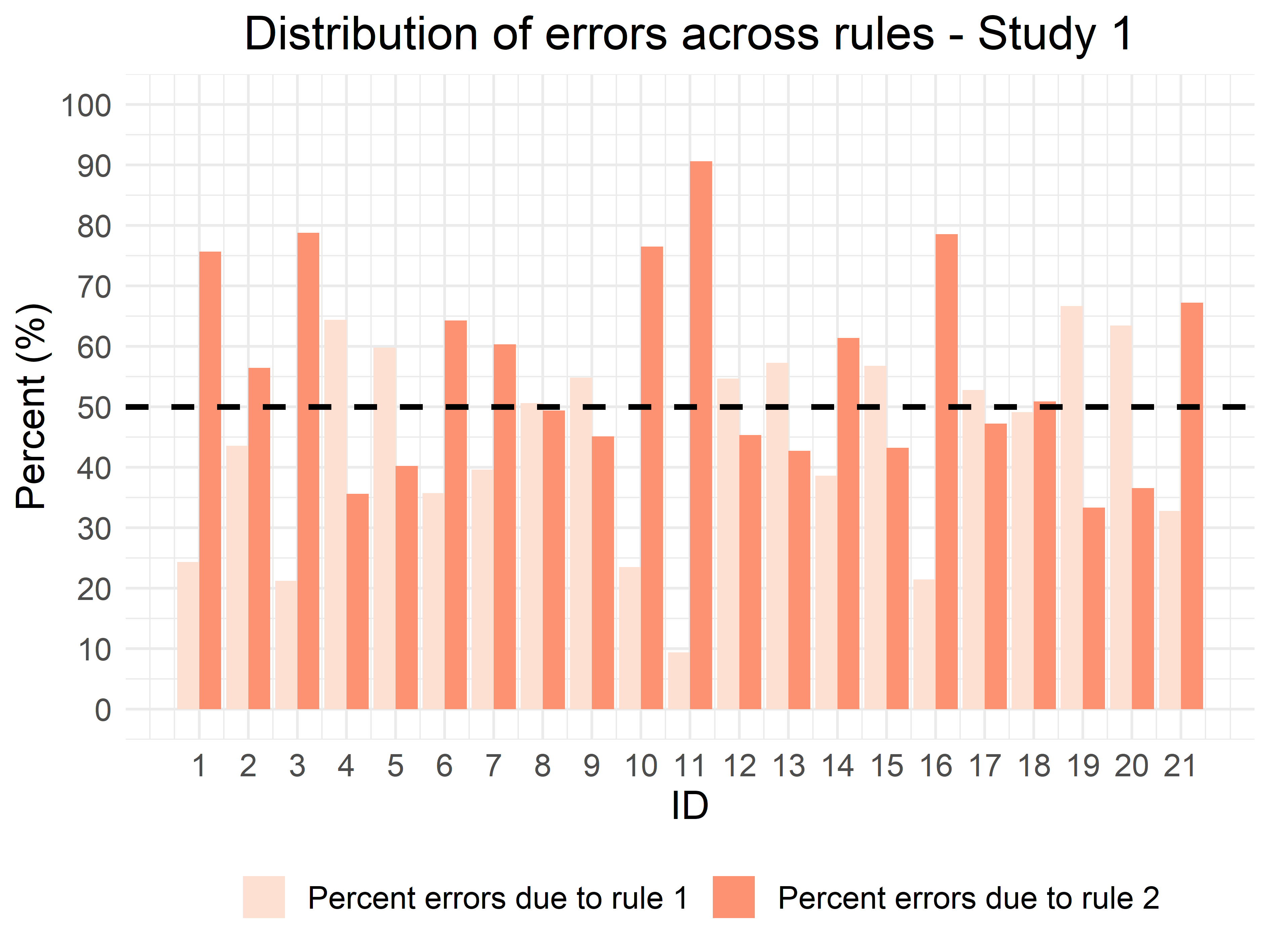


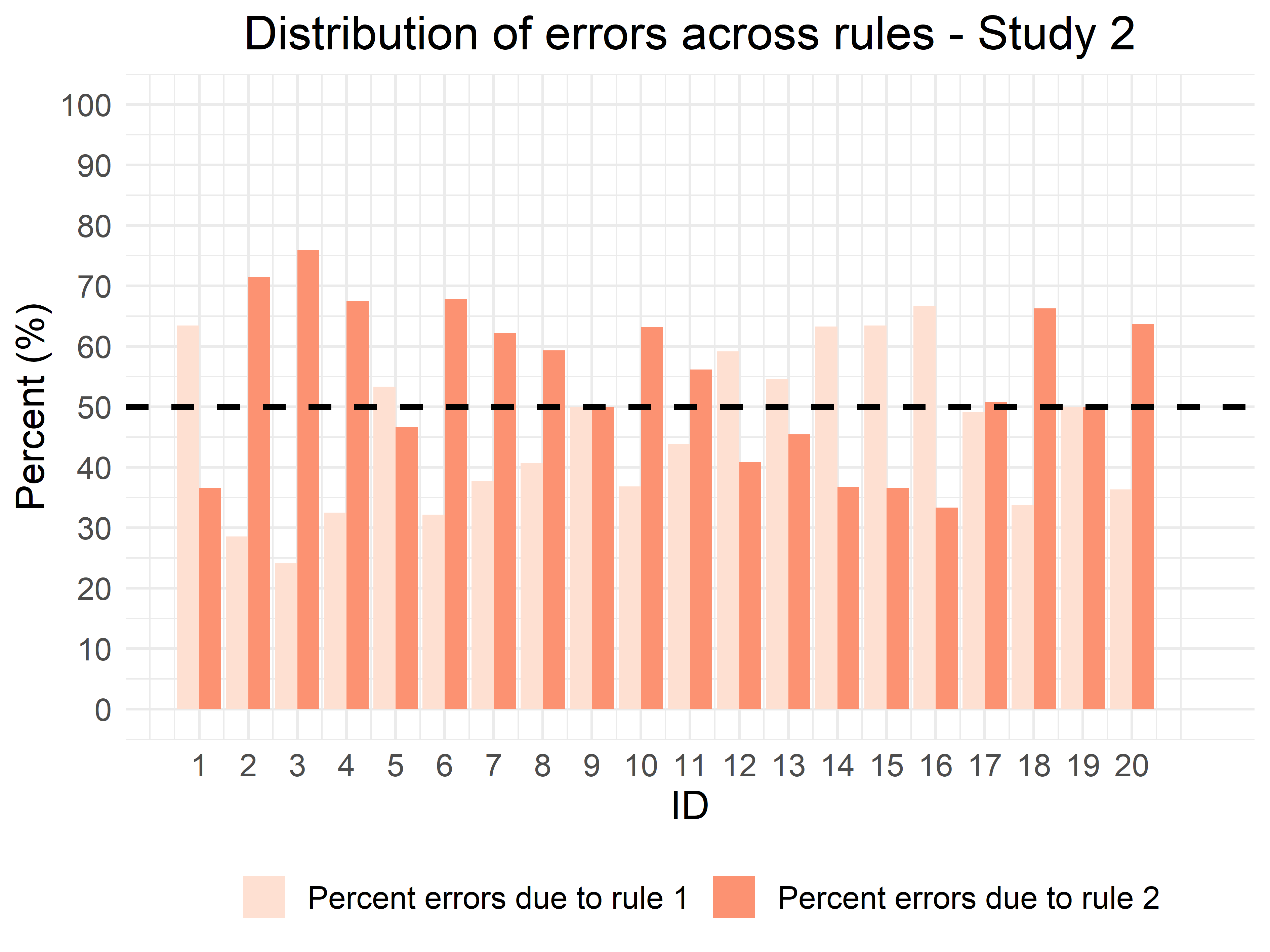


1. Additional results of study 2

*Influence of rule type in RTs*

Similar to RT results from study 1, we here again saw an influence of rule type on RTs. A 2x2 RM ANOVA with the factors rule and accuracy showed a main effect of rule [F(1,76) = 59.234, p < .001, η2G = .438] with slower RTs for rule 2 (1793.0 ± 291.6 ms) than for rule 1 (1348.2 ± 368.3 ms). Further, correct responses (1423.4 ± 441.5 ms) had faster RT compared to errors [1717.8 ± 287.8 ms; F(1,76) = 25.951, p < .001, η2G = .255]. However, the interaction between accuracy and rule was also significant [F(1,76) = 26.903, p < .001, η2G = .261]. Post-hoc tests revealed that RTs were fastest for correct responses on rule 1 (1051.2 ± 150.5 ms), followed by errors on rule 1 (1645.3 ± 264.6 ms) and errors and correct responses on rule 2 (1790.4 ± 298.2 ms; 1795.7 ± 292.6 ms, respectively; p > .70, all other ps < .001).

*Behavioural adjustments*

When analysing PES in a similar way as in study 1 (i.e., averaged across all blocks), we again found a significant difference between PES (47.9 ± 81.4 ms) and PCS [2.8 ± 17.4 ms; t(19) = 2.190, p = .041, d = .489], and no significant effect of error awareness on PES [PES for detected errors = 14.8 ± 104.7 ms; PES for undetected errors = 57.6 ± 177.6 ms; t(19) = -.832, p = .416, d = -.083].

The same was true for behavioural adjustments in terms of accuracy. Post-error accuracy (74.8 ± 8.0 %) was significantly different from the post-correct accuracy [77.7 ± 2.9 %; t(19) = -1.476, p = .156, d = .330]. Again, error awareness did not influence the post-error accuracy [PEA for detected errors = 76.1 ± 9.2 %; PEA for undetected errors = 74.2 ± 10.6 %; t(19) = 0.706, p = .489, d =.158].

Finally, we examined adjustments due to the occurrence of timeouts. As in study 1, we observed a speeding of RTs after timeouts compared to correct responses [PTS: -79.7 ± 114.0 ms; t(19) = -3.037, p = .007, d = -.679]. Likewise, more timeouts occurred after errors (9.99 ± 5.8 %) than after correct responses [7.33 ± 4.90 %; t(19) = 2.255, p = .036, d = .504]. Thus, all findings regarding behavioural adjustments from study 1 could be replicated by an independent sample in study 2.

*Rule-related errors – RT results*

In the main text, we showed that errors on trials where rule 1 should be applied could be differentiated into two error types depending on whether or not participants applied rule 2 on these trials. Whenever participants “correctly” applied rule 2, despite the fact that the hierarchically higher rule 1 was actually to be applied, this often resulted in undetected errors. In addition, these two error types were significantly different in terms of RTs (t(19) = -2.946, p = .008, d = -.659): Errors were on average faster (1573.5 ± 226.7 ms) when participants did not apply rule 2 compared to when they used the incorrect rule (1706.0 ± 272.6 ms). The latter RT corresponds well to the mean RT of correct responses on trials applying rule 2 (cf. 1790.4 ± 298.2 ms taken from the second study).
